# Scaling-up coral reef carbonate production: Sea-urchin bioerosion suppresses reef growth in Hawaiʻi

**DOI:** 10.1371/journal.pone.0324197

**Published:** 2025-05-28

**Authors:** Kelly J. van Woesik, Jiwei Li, Gregory P. Asner

**Affiliations:** 1 Center for Global Discovery and Conservation Science, Arizona State University, Hilo, Hawaiʻi, United States of America; 2 Center for Global Discovery and Conservation Science, Arizona State University, Tempe, Arizona, United States of America; 3 School of Ocean Futures, Arizona State University, Tempe, Arizona, United States of America; MARE – Marine and Environmental Sciences Centre, PORTUGAL

## Abstract

Coral reefs provide essential social, economic, and ecological services for millions of people worldwide. Yet, climate change and local anthropogenic stressors are damaging reefs globally, compromising their framework-building capacity and associated functionality. A reef carbonate budget provides a quantitative measure of growth and functional status, but utilization of remote sensing to scale-up such a metric remains limited. This study used census-based field surveys across depths in Hōnaunau Bay, Hawaiʻi to examine rates of carbonate production, and scaled-up estimates across the bay with high-resolution benthic-cover data derived from airborne imaging spectroscopy. Average net carbonate production was ~0.5 kg CaCO_3_ m^-2^ y^-1^ across the 2–17 m depth gradient, ranging from -2.1 to 2.4 kg CaCO_3_ m^-2^ y^-1^ at 3 and 6 m, respectively. The scaling model with the lowest root mean square error was achieved using a 2-m resolution map of live coral cover. Sea-urchin densities averaged 51 individuals m^-2^, which were among the highest recorded densities on coral reefs globally. The subsequent high bioerosion from sea urchins suppressed estimated reef-growth potential, particularly in the shallow reef <6 m. Field estimates of net carbonate production translate to vertical reef accretion of ~0.5 mm y^-1^ across depths, indicating the reef in its present form is not keeping pace with the current rate of sea-level rise (3.55 mm y^-1^) in west Hawaiʻi. These results suggest a need for improved fisheries management in Hōnaunau Bay to enhance carnivorous-fish abundances, thereby helping to reduce sea-urchin densities and improve reef-growth capacity. Critically, an estimated threshold of ~26% live coral cover is currently needed to maintain positive net production across depths. This study demonstrates the utility of monitoring carbonate production by integrating field measurements and airborne imaging spectroscopy, and highlights the need for management decisions in west Hawaiʻi that enhance resilient carbonate budgets of coral reefs.

## Introduction

Coral reefs support more than a quarter of all marine species [1] and provide essential geo-ecological functions for marine ecosystems, including reef-building capacity, sediment generation, and habitat complexity [2]. Such functionality also underpins the essential goods and services that reefs provide to more than a billion people worldwide, such as food sources and barriers against coastal erosion by storm waves [3,4]. Yet, climate change [5] and local anthropogenic stressors [6] are damaging coral reefs globally and changing their essential functions [7]. Indeed, reef degradation jeopardizes the capacity of reefs keep pace with sea-level rise and protect shorelines [4,8].

Growth of coral reefs depends upon the production and maintenance of the reef framework through accumulation of calcium carbonate (CaCO_3_) [9]. Together, the growth and erosion of a reef drives its carbonate budget, where corals, coralline algae, and sediment contribute to reef growth, and chemical (e.g., ocean acidification), physical (e.g., hurricanes), and biological processes (e.g., boring organisms, echinoids, and fishes) contribute to reef erosion [10–13]. Carbonate production can be further reduced by land-use changes and runoff of terrestrial sediment [14], in addition to thermal stress from marine heatwaves [15], which induce coral bleaching and mortality, reductions in coral densities, and changes in the composition of coral assemblages [5]. Carbonate production can also be suppressed by high rates of bioerosion, and several studies have shown that fishing pressure increases the number of bioeroding sea urchins on reefs [16–18]. Additionally, submarine groundwater discharge, which can contain high nutrients and low pH, can magnify the effects of ocean acidification, leading to decreased reef calcification, dissolution of the reef in extreme cases, and increased rates of bioerosion [19]. Together, these changes reduce reef integrity and the capacity of reefs to grow vertically and keep pace with sea-level rise [2].

Census-based carbonate budgets estimate carbonate production directly from biological growth and bioerosion [9]. Considering that reef-accretion potential and structural maintenance are largely biologically driven [13,20], census-based estimates provide a ‘snap-shot’ of ecological processes from days to years [9] and thus a metric of functional-reef performance [21]. There is growing interest in using census-based estimates of carbonate production as a diagnostic and predictive tool for quantifying reef accretion and shoreline responses under a changing climate [9]. There is also a need for an increased understanding of the interactions between carbonate production and bioerosion across reef habitats and depths — as most studies have previously focused only on shallow reefs < 10 m [2,21] — in addition to how essential geo-ecological functions of healthy reefs can be maintained and restored [2].

Despite these recognized needs, census-based field sampling remains time consuming and spatially limited [21]. Remote sensing, by contrast, can survey large geographic areas [22,23], and recent advances in satellite sensors have facilitated mapping of coral-reef extent, benthic features, and geomorphic zones [23]. While most reef classifications are based on these broad categories, advances in airborne sensors, like the Global Airborne Observatory (GAO; [24]), have facilitated mapping of benthic cover using imaging spectroscopy [25]. Combining field sampling with remote-sensing technology may provide the pathway needed to estimate carbonate budgets at broad spatial scales. Carbonate production has previously been estimated using satellite imagery of benthic communities in Australia [26,27]; however, those estimates did not consider bioerosion rates within the carbonate budget. Airborne imaging spectroscopy has previously been used to scale-up estimates of coral chlorophyll concentrations [28] and thermal tolerance [29], but it has not yet been used to scale-up estimates of carbonate production.

This study seeks to provide a first estimate of net carbonate production across depths on Hawaiʻi Island. Census-based field estimates and high-resolution benthic-cover data — derived from airborne imaging spectroscopy collected via the Global Airborne Observatory [25] — were utilized to scale-up estimates of carbonate production. Such an approach has the potential to enhance monitoring of carbonate production over large spatial and temporal scales to determine where coral reefs are potentially keeping up with anthropogenic stressors, ocean warming, and sea-level rise. The objectives of this work were to: 1) estimate rates of carbonate production in Hōnaunau Bay, Hawaiʻi Island across depths, 2) determine the percent of live coral cover when rates of carbonate production become negative (i.e., from high bioerosion rates), and 3) utilize field data to scale-up estimates of carbonate production using benthic-cover data derived from airborne imaging spectroscopy.

## Materials and methods

### Study site

**Fig 1:**
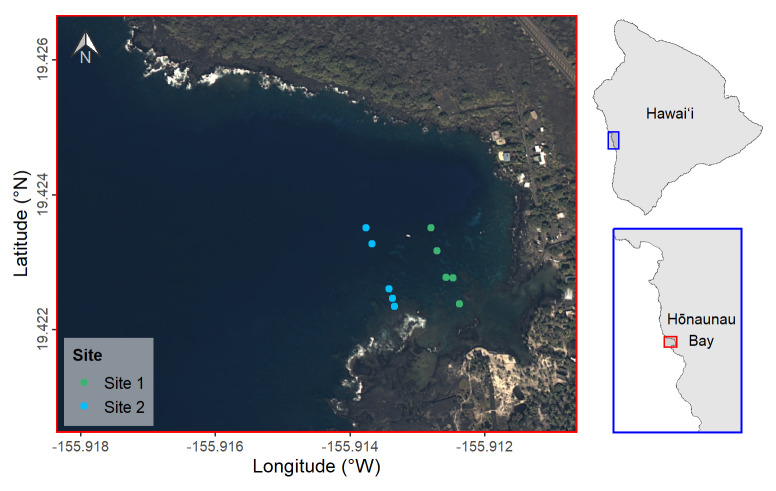
Locations of field sampling at two sites in Hōnaunau Bay, Hawai'i Island, 2023. Insets illustrate the bay’s location relative to Hawaiʻi Island. Coordinates are located in Table F in S1 File. Background image: USGS NAIP Plus from The National Map.

This study was conducted in Hōnaunau Bay, located on the south-central, leeward (western) coast of Hawaiʻi Island. Hydrographically, it is a typical embayment found in Hawaiʻi and supports corals to 20 m [30]. The bay is impacted by three main stressors, which include heating events, fishing pressure, and water pollution; these stressors are common in Hawaiʻi [6]. To estimate carbonate production across depths, surveys were conducted across the natural depth gradient at 2 m, 3 m, 6 m, 9 m, and 17 m below mean-sea level (MSL). To reduce potential confounding variables associated with surveying different sides of the bay, two sites were randomly selected to capture a representative sample of the bay’s south side (Fig 1).

### Experimental design and field surveys

To derive accurate estimates of reef carbonate production for Hōnaunau Bay, detailed field surveys were conducted using a census-based technique, following a modification of the ReefBudget methodology adapted for the Indo-Pacific [31]. This method combines field estimates of benthic cover, reef rugosity, and bioerosion rates by sea urchins and herbivorous fishes. Field surveys were conducted in May 2023, and did not involve any endangered species. Appreciation is extended to the indigenous stewards of Hōnaunau Bay, who non-financially supported the research.

Due to high sea-urchin densities observed in preliminary field observations at Hōnaunau Bay, small 0.25 m^2^ quadrats were utilized for urchin surveys [32–35]. At each depth (2, 3, 6, 9, and 17 m) across the two sites, densities of sea urchins were recorded within 15 randomized quadrats (for a total of 150 quadrats surveyed overall). Quadrat 1 was placed under each Global Positioning System (GPS) coordinate, and the remaining 14 quadrats for each site and depth were haphazardly placed in the surrounding area so tapes in benthic surveys would be approximately 1–2 m apart, while ensuring a similar depth. In each quadrat, sea urchins were identified in the field to species level and test (i.e., skeleton) size was measured to the nearest half cm. Each quadrat was also photographed using a Sony RX100M5 camera in an underwater housing. The optimal number of replicates needed (n = 15) to minimize field time while capturing sea-urchin variation in quadrats was determined from a pilot study undertaken in Hōnaunau Bay where 23 quadrats were sampled at 3 m depth and 29 quadrats were sampled at 6 m depth. Following [36], the coefficient of variation (CV) was used to select the optimal replicate number based on where the CV plateaued (Fig A in S1 File). The most-abundant sea urchin observed, *Echinometra mathaei*, was used in the CV analysis (Figs B**–**E in S1 File).

To match the benthic surveys to the pixel size of remote-sensing data, a 2-m tape (tape 1) was laid horizontally across the reef substrate, centered under each quadrat (n = 15). A second tape (tape 2) was placed under the first tape, along the top of the reef surface, following the contours of the reef. Using tape 2 (the sampling transect), a modified line-intercept method was used to record the benthic composition every cm [31], recording corals to species level and other taxa to the highest possible resolution. The length of the contoured transect of tape 2 (i.e., the cumulative total reef surface) was divided by the 2-m linear distance of tape 1 to quantify rugosity, a measure of topographical complexity [31]. The depth at the center of each quadrat (and thus transect) was recorded and adjusted to depth below MSL (Table A in S1 File), so the data could be comparable to the MSL depths recorded by remote-sensing data.

Parrotfishes are the main eroding group of herbivorous fishes on reefs, classified as reef scrapers and excavators; therefore, census-based budget methods also focus on quantifying erosion rates of parrotfishes [31]. At each depth (2, 3, 6, 9, and 17 m) across the two sites, fishes were videoed using a Canon VIXIA HFM500 video camera in an underwater housing along six 25 x 5 m belt transects (for a total of 60 transects surveyed overall). Six is a common replicate recommended for use in census-based budget estimates [37] and 25 x 5 m is a standard transect length utilized in Hawaiʻi [38–41]. For each transect, the density of parrotfishes was subsequently recorded to species level and the length of each individual was estimated to the nearest cm.

### Estimating net carbonate production

Net carbonate production (kg CaCO_3_ m^-2^ y^-1^) in the field was estimated to the transect level, *i*, using the following equation [42]:


$$Net\;carbonate\;production_{i}=gross\;carbonate\;production_{i}\;+\;sgn\left(x\right)sedimentation-bioerosion_{i}$$ (1)

where *gross carbonate production* is the calcification rate of reef-building organisms in transect *i*; *sedimentation* is the rate of sediments contributing to reef accretion in the bay, which increases carbonate production when sedimentation rates are low (< 0.05 kg m^-2^ d^-1^) [42,43], making *sgn(x)* positive, and decreases carbonate production when sedimentation rates are high, due to smothering of corals by terrestrial sediment [14], making *sgn(x)* negative; and *bioerosion* is the rate of biological erosion in transect *i*. Net carbonate production values at the site and depth level, and for each depth, were estimated by averaging the transect values.

### Estimating gross carbonate production

Gross carbonate production (kg CaCO_3_ m^-2^ y^-1^) from calcifying reef taxa in transect *i* was estimated using the following equation:


$$Gross\;carbonate\;production_{i}=r_{i}\left[\sum \left(x_{i}+ca_{i}+h_{i}\right)\right]$$ (2)

where *r* is the rugosity of transect *i*; *x* is the calcification rate of corals in transect *i*; *ca* is the calcification rate of coralline algae in transect *i*; and *h* is the calcification rate of *Halimeda*, a calcareous green macroalgae, in transect *i*.

In Equation (2), the contribution of reef-building corals (*x*) to carbonate production (kg CaCO_3_ m^-2^ y^-1^) in transect *i* was estimated using the following equation:


$$x_{i}=\;\sum \left(m_{i,j}*px_{i,j}*d_{i,j}*g_{i,j}*10\right)$$ (3)

where *m* is the adjustment coefficient for coral morphology for species *j* in transect *i* based on morphologies for Indo-Pacific corals ([44]; Table B in S1 File); *px* is the planar proportion of coral species *j* in transect *i*; *d* is the skeletal density (g cm^-3^) of coral species *j* in transect *i* based on averaged values to the genus/morphology level from studies across depths in the Indo-Pacific ([31]; Table C in S1 File); *g* is the vertical growth rate (cm y^-1^) of coral species *j* in transect *i* based on values to the species level across depths in Hawaiʻi when available, otherwise averaged to the genus/morphology level from studies in the Indo-Pacific (Table D in S1 File); and 10 is the conversion factor to kg m^-2^ y^-1^.

As this study assessed carbonate production across depths, the vertical growth rates of coral species (*g*) in Equation (3), above, were adjusted according to sampling depth, when such rates were available in the literature. Growth rates for *Porites* corals in this study (cm y^-1^) were estimated following equations in [45] for *Porites lobata* off Maui, Hawaiʻi using the following linear growth-rate equation for depths < 6 m:


$$g_{i,j}=\frac{\left(13.37-0.21*depth\right)}{10}$$ (4)

and the following exponential growth-rate equation for depths ≥ 6 m:


$$g_{i,j}=\frac{16.2e^{-0.032*depth}}{10}$$ (5)

where 10 was used to convert growth rates from mm y^-1^ to cm y^-1^. For all other coral-growth rates used in this study, values were averaged to the species or genus/morphology level, preferably from studies in Hawaiʻi, otherwise averaged across studies in the Indo-Pacific [31], where depths were either not specified or were from depths < 8 m (see Table D in S1 File for more details). Due to the low abundance of non-*Porites* corals surveyed in this study (Fig F in S1 File), the lack of non-*Porites* growth rates across depths was assumed to have a minimal impact on the overall estimates of calcification.

In Equation (2), the contribution of coralline algae (*ca*) to carbonate production (kg CaCO_3_ m^-2^ y^-1^) in transect *i* was estimated using the following equation:


$$ca_{i}=0.036*pca_{i}*10$$ (6)

where 0.036 is the average calcification rate of coralline algae (g cm^-2^ y^-1^) in shallow-water reefs in the central Pacific Ocean [31]; *pca* is the planar proportion of coralline algae in transect *i*; and 10 is the conversion factor from g cm^-2^ y^-1^ to kg m^-2^ y^-1^. Similar to non-*Porites* corals, due to the low abundance of coralline algae surveyed in this study, particularly at depth (Fig F in S1 File), the lack of calcification rates across depths was assumed to have a minimal impact on the overall estimates of calcification.

In Equation (2), the contribution of *Halimeda* (*h*), a calcareous green macroalgae, to carbonate production (kg CaCO_3_ m^-2^ y^-1^) in transect *i* was estimated using the following equation:


$$h_{i}=\;1.694*ph_{i}$$ (7)

where 1.694 is the average calcification rate of *Halimeda* (kg m^-2^ y^-1^) at 20 m depth off Oʻahu and the Maui-Nui complex, Hawaiʻi [46]; and *ph* is the planar proportion of *Halimeda* in transect *i*. Note that *Halimdea* was only recorded in transects at 17 m depth in this study, so the calcification rate of *Halimeda* at 20 m depth from [46] was used in Equation (7).

#### Sedimentation.

As outlined in the section above, carbonate sediment can influence reef accretion [14]. No obvious sedimentation from terrestrial sources was observed during the time of surveys in Hōnaunau Bay. Furthermore, long-term monitoring of nearshore turbidity does not include substantial sedimentation impacts in Hōnaunau Bay [47]. Therefore, calcareous sedimentation was considered to positively contribute to carbonate production in this study. A rate of 0.53 kg CaCO_3_ m^-2^ y^-1^ was assumed from estimates off Oʻahu, Hawaiʻi [48], derived from bioerosion and direct physical sources.

#### Estimating bioerosion.

Reef bioerosion (kg CaCO_3_ m^-2^ y^-1^) in transect *i* was estimated using the following equation:


$$Bioerosion_{i}=\sum \left(parrotfish_{k}+sea\;urchin_{i,j}+marcoboring_{i}+microboring_{i}\right)$$ (8)

where *parrotfish* is the biological erosion rate from parrotfishes at the site and depth *k*; *sea urchin* is the biological erosion rate of sea-urchin species *j* in quadrat *i*; *macroboring* is the biological erosion rate of macroboring organisms in transect *i*; and *microboring* is the biological erosion rate of microboring organisms in transect *i*. Note that only one *Acanthaster planci*, a coral predator, was observed during surveys, so incorporating its effects on bioerosion was not considered in this study.

#### Estimating bioerosion from parrotfishes.

In Equation (8), the contribution of *parrotfish* to bioerosion (kg CaCO_3_ m^-2^ y^-1^) at each site and depth *k* was estimated from the average values across transects using the following equation:


$$parrotfish_{k}=\frac{\sum \left(parrotfish_{i}\right)}{6}$$ (9)

where 6 is the number of parrotfish transects at the site and depth *k*; and *parrotfish*_*i*_ is the parrotfish bioerosion (kg CaCO_3_ m^-2^ y^-1^) in transect *i*, estimated using the following equation:


$$parrotfish_{i}=\sum \left(parrotfish_{i,j}\right)$$ (10)

where *parrotfish*_*i,j*_ is the parrotfish bioerosion (kg CaCO_3_ m^-2^ y^-1^) for each species *j* in transect *i*, estimated using the following equation:


$$parrotfish_{i,j}=\sum \left(\frac{vol_{i,j,n}*sp_{i,j,n}*br_{i,j,n}*D_{k}*365*0.001}{25*5}\right)$$ (11)

where 25 x 5 is the length and width in meters of the fish transect *i*; *vol* is the volume of a single bite (cm^-3^) for parrotfish individual *n* of species *j* in transect *i*; sp is the proportion of bites that leave a scar for parrotfish individual *n* of species *j* in transect *i*; *br* is the bite rate (bites day^-1^) for parrotfish individual *n* of species *j* in transect *i*; *D* is the average density of coral skeletons (g cm^-3^) for the site and depth *k*; 365 is the conversion factor from days to years; and 0.001 is the conversion factor from g to kg.

In Equation (11), bite volume (*vol*) was estimated using the following equation:


$$vol_{i,j,n}=\frac{e^{1.3172+0.0624*length_{i,j,n}}}{1000}\;$$ (12)

where *length* is the length in cm for parrotfish individual *n* of species *j* in transect *i*; 1.3172 and 0.0624 are constants (derived from [49]); and 1000 is the conversion factor from mm^3^ to cm^3^.

In Equation (11), the proportion of bites leaving scars (*sp*) was estimated using the following equation:


$$sp_{i,j,n}=\frac{1}{1+e^{-\left(-2.46142+0.08864*length_{i,j,n}\right)}}\;$$ (13)

where *length* is the length in cm for parrotfish individual *n* of species *j* in transect *i*; and the equation is based on regression data (derived from [49,50]).

In Equation (11), the bite rate (*br*) was estimated using the following equation:


$$br_{i,j,n}=60\left[\left|\left(4.31+brc_{i,j,n}-0.355\right)-\left(0.045*bitetime*length_{i,j,n}\right)\right|\right]\;$$ (14)

where *length* is the length in cm for parrotfish individual *n* of species *j* in transect *i*; *bitetime* is the length of time parrotfishes spend grazing on the reef, where 10 hours a day was assumed [50]; 60 is the conversion factor from minutes to hours; *brc* is the bite rate constant for parrotfish individual *n* of species *j* in transect *i* based on data from the Indo-Pacific to the species level when available, otherwise to the sister-species level based on feeding similarities (Table E in S1 File), with other constants derived from the bite-rate data [44].

#### Estimating bioerosion from sea urchins.

In Equation (8), the contribution of echinoid (*sea urchin*) species *j* to bioerosion (kg CaCO_3_ m^-2^ y^-1^) in quadrat *i* was estimated using the following equation:


$$sea\;urchin_{i,j}=\;\sum \left(Diadematidae_{i,j,n}+Echinometra_{i,j,n}+Other\;eroding\;sea\;urchins_{i,j,n}\right)$$ (15)

where Diadematidae is the biological erosion rate for sea-urchin individual *n* of species *j* within the genus *Diadema* or *Echinothrix* in quadrat *i*; *Echinometra* is the biological erosion rate for sea-urchin individual *n* of species *j* within the genus *Echinometra* in quadrat *i*; and *Other eroding sea urchins* is the biological erosion rate for sea-urchin individual *n* of other eroding species *j* in quadrat *i*, which includes all other sea-urchin species surveyed in this study, except for the non-eroding species *Tripneustes gratilla* [31].

In Equation (15), size-specific bioerosion rates of the three categories of sea urchins were estimated using equations from published studies in the Indo-Pacific [31], and input into equations following [51]. Specifically, the contributions of sea urchins to bioerosion (kg CaCO_3_ m^-2^ y^-1^) in transect *i* were estimated using the following equations:


$$Diadematidae_{i,j,n}=\;\frac{\sum \left[\left(0.000003*diameter_{i,j,n}^{\;3.2887}\right)*0.365*0.57\right]}{0.25}$$ (16)

$$Echinometra_{i,j,n}=\;\frac{\sum \left[\left(0.000300*diameter_{i,j,n}^{\;1.9671}\right)*0.365*0.57\right]}{0.25}$$ (17)

$$Other\;eroding\;sea\;urchins_{i,j,n}=\;\frac{\sum \left[\left(0.000030*diameter_{i,j,n}^{\;2.6414}\right)*0.365*0.57\right]}{0.25}$$ (18)

where 0.25 is the surface area of quadrat *i* in m^2^; *diameter* is the diameter in mm of the test for sea-urchin individual *n* of category *j* in quadrat *i*; 0.365 is the conversion factor from g sea urchin^-1^ day^-1^ to kg sea urchin^-1^ yr^-1^; and 0.57 is a correction factor added to account for the proportion of sediment re-ingested during grazing [52].

#### Estimating macro- and microbioerosion.

In Equation (8), the contribution of endolithic *macroboring* organisms (e.g., clinoid sponges, polychaete worms, bivalves, molluscs) to bioerosion (kg CaCO_3_ m^-2^ y^-1^) in transect *i* was estimated using the following equation:


$$macroboring_{i}=r_{i}*0.21*pmacro_{i}$$ (19)

where *r* is the rugosity of transect *i*; 0.21 is the average macrobioerosion rate for reefs between 1–18 m depth in the central Pacific Ocean [31]; and *pmacro* is the planar proportion of substrate available for macrobioerosion in transect *i*. Substrate available for macrobioerosion includes carbonate substrate and rubble (including that overgrown by algae), in addition to coralline algae because even thick crustose coralline algae does not prevent macrobioerosion of the underlying substrate [31].

In Equation (8), the contribution of endolithic *microboring* organisms (e.g., cyanobacteria, chlorophytes, and fungi) to bioerosion (kg CaCO_3_ m^-2^ y^-1^) in transect *i* was estimated using the following equation:


$$microboring_{i}=r_{i}*0.20*pmicro_{i}$$ (20)

where *r* is the rugosity of transect *i*; 0.20 is the average microbioerosion rate for reefs between 1–18 m depth in the central Pacific Ocean [31]; and *pmicro* is the planar proportion of substrate available for microbioerosion in transect *i*. Substrate available for microbioerosion includes carbonate substrate and rubble (including that overgrown by algae), whereas benthic categories with a net calcification rate that already includes rates of microbioerosion (i.e., corals and coralline algae) were excluded [31].

### Estimating vertical reef accretion

To convert net carbonate production estimated in Equation (1) to vertical reef accretion (mm), the following equation was used:


Vertical reef accretion=Cp+Cp[Cp*(−0.01949)] (21)

where *Cp* is net carbonate production and -0.01949 is a coefficient estimated for the best-fit function (following [44]). Vertical reef-accretion values at the site and depth level, and for each depth, were estimated from the average net-production values.

### Data analysis of field estimates

All statistical analyses were conducted in R version 4.3.2 [53]. Linear mixed-effects models, run using the *lme4* package [54], were used to determine changes in: (i) net carbonate production, (ii) gross carbonate production, (iii) bioerosion, (iv) sea-urchin abundance, and (v) live coral cover across depths, where depth was a fixed effect and site (i.e., the two randomly-selected sites comprising the depth gradients) was a random effect formatted as a random-intercept model. Tukey post-hoc tests, run using the *emmeans* package [55], were then used to assess the significant differences between pairs of means for depths.

To determine the percent of live coral cover when rates of carbonate production became negative at each depth, an additive mixed-effects model was used in a Bayesian framework following [56] — who used a similar approach in the Caribbean — following the equation:


$$Net\;carbonate\;production_{i,s}=Beta+f\left(Live\;coral\;cover_{i,s}\right)+Depth_{i,s}+a_{s}+error_{i,s}$$ (22)

where *net carbonate production* is the value from transect *i* at site *s*; *Beta* is a regression parameter; *f(Live coral cover)* is an O’Sullivan spline smoothing function [57] with 5 knots [56]; *Depth* is the fixed effect of depth; and *a* is a random intercept, using a normal distribution, for the site *s*; and *error* is the term for residuals. Multivariate-normal-diffuse and normal-diffuse priors were employed because an assumption was made that no prior information was known for the analysis. The models were run using JAGS via the *rjags* package in R [58].

### Scaling-up field estimates of carbonate production using remote sensing

Airborne imaging spectroscopy data were collected by the Global Airborne Observatory (GAO), formerly known as the Carnegie Airborne Observatory [24]. A detailed description of the GAO coral-mapping and processing procedures can be found in [25], and the GAO rugosity and bathymetry mapping and processing can be found in [30,59]. In brief, two coaligned instruments were used for mapping, including a high-fidelity visible-to-shortwave infrared (VSWIR) imaging spectrometer with a 5 nm resolution, and a dual-beamed light detection and ranging (LiDAR) scanner with a 60-megapixel camera. Benthic reflectance, and water depth relative to MSL, were retrieved by applying an atmospheric correction model, removing glint, and orthorectifying flight lines, which were conducted between 08:30 and 11:00 Hawaiian standard time. Rugosity was derived from maps of bathymetry built from the spectroscopy data, and a neural-network model was utilized to classify VISWIR reflectance data into the percentage cover of live coral, algae, and sand, where algae is defined as both turf and macroalgae [25].

Mapping of bathymetry and rugosity to a 2-m spatial resolution was conducted in 2019 and 2020 [30,59], and the data are publicly available online (bathymetry: https://zenodo.org/records/4294324 [60]; rugosity: https://zenodo.org/records/4294332 [61]). Mapping of live coral cover to a 2-m spatial resolution was conducted in 2019 [25] and in 2020 [62], with the 2019 campaign comprising extensive field validation; both years of data are publicly available online (2019: https://zenodo.org/records/4292660 [63]; 2020: https://zenodo.org/records/4777345 [64]). Mapping of benthic categories to 20 m depth took place again in January 2023, employing a similar classification model from 2019 [25] with field validation, and was utilized in this study.

To scale-up estimates of net carbonate production using remote sensing for this study, firstly, relationships were examined among field data. The field variables of percent live coral cover, depth (m), rugosity, and percent substrate available for algal growth (i.e., carbonate, rubble, coralline algae, and *Halimeda*) were used to match available airborne data. A partial least squares regression (PLSR), with k-fold cross-validation, was run using the *pls* package [65] and used to determine the optimal number of components to include in the model to estimate net carbonate production. Also, pairwise correlations were generated among the four field variables and collinear variables were determined using a cutoff value of ± 0.7 because values between ± 0.7 and ± 1 indicate a strong (±) linear relationship [66]. Collinear variables were identified and removed to prevent model overfitting. Outliers of net carbonate production were removed using a threshold of three times the mean of Cook’s distance. Then, a standard least-squares linear regression was run on the training data (70%) to predict net carbonate production (i.e., the dependent variable) from the independent variables remaining after testing for collinearity. Variables that did not significantly contribute to estimates of net carbonate production were removed and the models were re-run. Performance was determined by running models on the testing data (30%) and assessing the root mean square error (RMSE) and R² values. As a reminder, field data were collected at the same spatial resolution (2 m) as the GAO data to facilitate scaling. The most optimal linear model — which showed the lowest RMSE and strongest relationship between field estimates of net carbonate production as the response variable — was then re-run with all field data, excluding outliers, to predict net carbonate production for Hōnaunau Bay from GAO-high-resolution data.

## Results

### Field estimates of carbonate production and reef accretion

**Fig 2:**
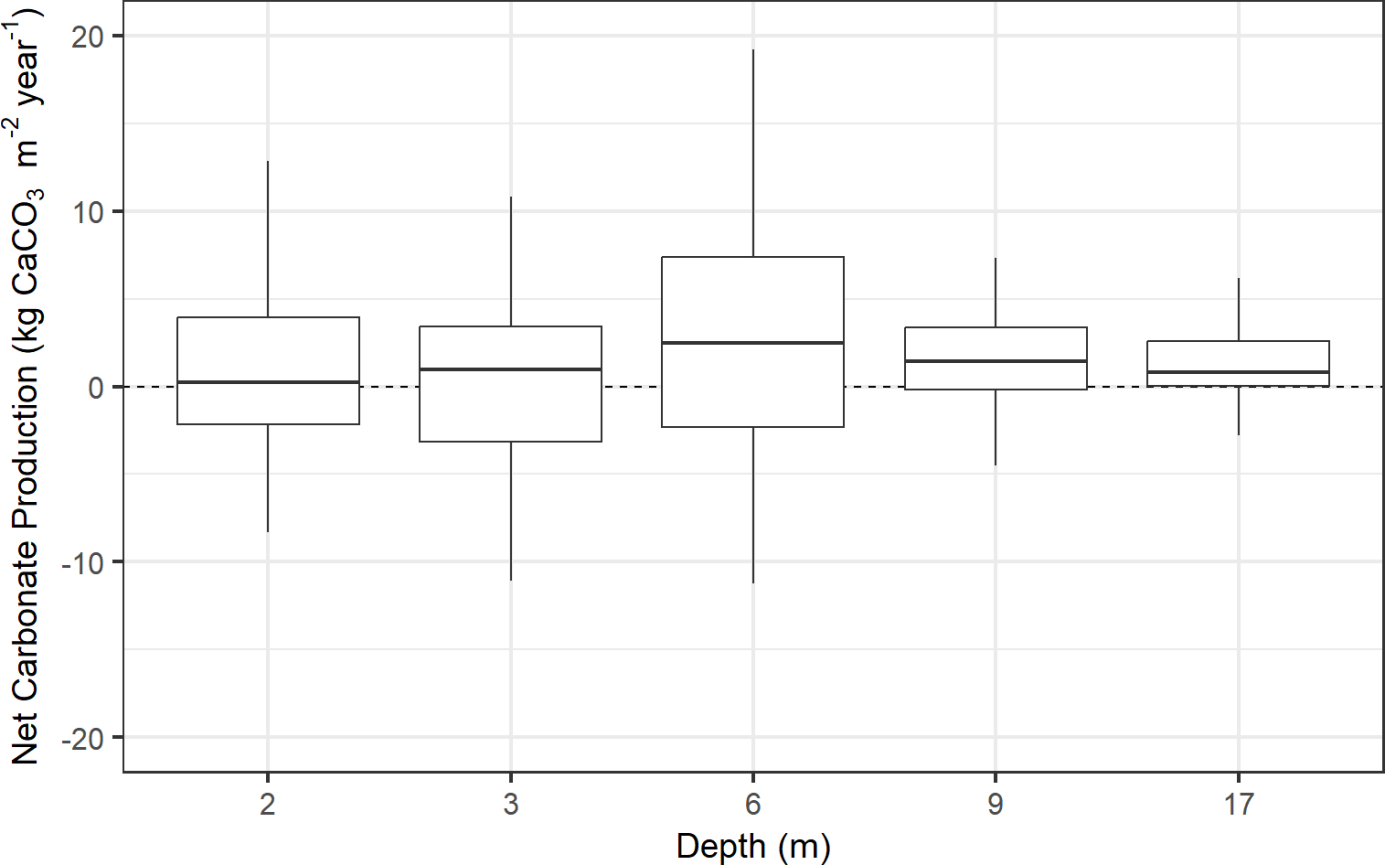
Net carbonate production rates of the reef (kg CaCO_3_ m^-2^ yr^-1^) at Sites 1 and 2 across depths (2–17 m) in Hōnaunau Bay, Hawai'i Island, 2023. Net carbonate production is gross carbonate production plus sedimentation minus bioerosion rates. The thick horizontal lines indicate medians, the boxes indicate the first and third quartiles, and the whiskers indicate the range of the data. Note that outliers were removed from the display. A linear mixed-effects model and Tukey post-hoc test showed no significant differences in net carbonate production among depths.

Net carbonate production was found to be, on average, 0.2 kg CaCO_3_ m^-2^ y^-1^ at 2 m, net negative (-2.1 kg CaCO_3_ m^-2^ y^-1^) at 3 m, 2.4 kg CaCO_3_ m^-2^ y^-1^ at 6 m, 1.3 kg CaCO_3_ m^-2^ y^-1^ at 9 m, and 1.0 kg CaCO_3_ m^-2^ y^-1^ at 17 m, with an average rate of 0.5 kg CaCO_3_ m^-2^ y^-1^ across depths (Fig 2 and Table 1). This translates into a vertical reef-accretion capacity of approximately 0.5 mm y^-1^ across depths, 0.2 mm y^-1^ at 2 m, -2.2 mm y^-1^ at 3 m, 2.3 mm y^-1^ at 6 m, 1.2 mm y^-1^ at 9 m, and 1.0 mm y^-1^ at 17 m (Table 1 and Fig G in S1 File). Carbonate-budget values split for each site and depth are displayed in Fig H and Table F in S1 File, and for each individual transect in Table G in S1 File.

**Table 1:** Coral reef carbonate budgets at Sites 1 and 2 across depths (2–17 m) in Hōnaunau Bay, Hawai'i Island, 2023.

Depth (m)	Gross Production (kg CaCO_3 _m^-2^ y^-1^)	Bioerosion (kg CaCO_3 _m^-2^ y^-1^)	Net Production (kg CaCO_3 _m^-2^ y^-1^)	Reef- Accretion Potential (mm y^-1^)
2	4.84 ± 0.61	-5.19 ± 0.86	0.17 ± 0.97	0.17 ± 1.04
3	5.67 ± 0.67	-8.31 ± 1.63	-2.11 ± 1.89	-2.20 ± 2.76
6	7.03 ± 0.87	-5.16 ± 0.76	2.40 ± 1.36	2.29 ± 1.25
9	3.65 ± 0.42	-2.93 ± 0.48	1.25 ± 0.68	1.22 ± 0.71
17	2.85 ± 0.57	-2.39 ± 0.75	0.99 ± 1.00	0.97 ± 1.13
Average	4.81 ± 0.31	-4.80 ± 0.46	0.54 ± 0.57	0.53 ± 0.70

Gross production is the rate of carbonate production, excluding sedimentation and bioerosion rates. Bioerosion is the rate of erosion from sea urchins, parrotfishes, macrobioerosion, and microbioerosion. Net production is gross carbonate production plus sedimentation minus bioerosion rates. Net production translates to reef-accretion potential using Equation (21). Mean values across depths ± standard error are displayed.

**Fig 3:**
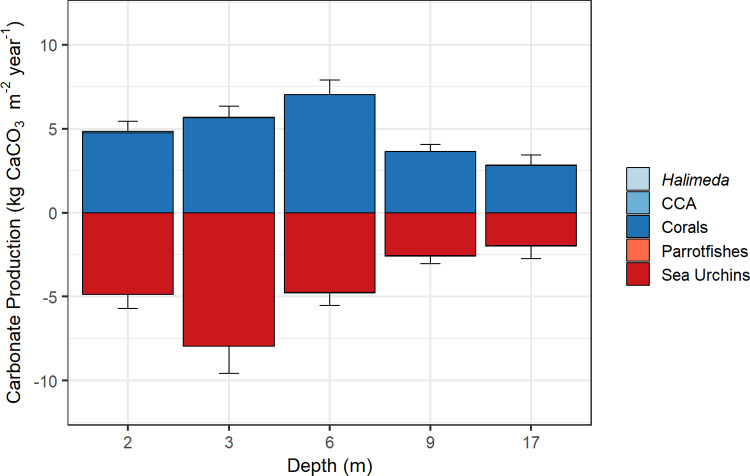
Gross carbonate production of calcifying benthic taxa (above zero) and bioerosion by eroding group (below zero) (kg CaCO_3_ m^-2^ yr^-1^) at Sites 1 and 2 across depths (2–17 m) in Hōnaunau Bay, Hawai'i Island, 2023. Mean values for each group across depths ± the standard error for the gross production (depicted in blue) or erosion (depicted in red) are displayed. Corals and sea urchins were the main contributors to gross carbonate production, whereas the contributions of *Halimeda*, crustose coralline algae (CCA), and parrotfishes were near negligible. Constants used in the carbonate-production estimates (i.e., sedimentation, macrobioerosion, and microbioerosion) are not displayed.

A linear mixed-effects model and Tukey post-hoc test showed no significant differences in net carbonate production among depths in Hōnaunau Bay, Hawaiʻi (Fig 2). However, significant variations among depths were apparent in the biological contributors of calcification and erosion (Fig 3). A linear mixed-effects model and Tukey post-hoc test showed that gross carbonate production was significantly lower (p ≤ 0.02) at 17 m than at 3 m and 6 m, and at 9 m versus 6 m (Fig I in S1 File). Gross carbonate production was mainly driven by corals (Fig 3), specifically *Porites lobata* (Fig J in S1 File), and *Porites* was the dominant genus on the reef (Fig F in S1 File). Average gross production across all depths combined was ~4.8 kg CaCO_3_ m^-2^ y^-1^, with the lowest rate of ~2.9 kg CaCO_3_ m^-2^ y^-1^ at 17 m, and the highest rate of ~7 kg CaCO_3_ m^-2^ y^-1^ at 6 m depth (Table 1).

A linear mixed-effects model and Tukey post-hoc test showed that bioerosion was significantly higher (p ≤ 0.001) at 3 m than at 9 m and 17 m (Fig K in S1 File). Biological erosion was mainly driven by sea urchins since bioerosion from parrotfishes was near zero (Fig 3 and Fig J in S1 File). Five parrotfish species were observed in surveys, where *Chlorurus spilurus* was the dominant species (Figs L and M in S1 File). Eleven sea-urchin species were observed, where *Echinometra mathaei* was the most abundant species, followed by the genus *Heterocentrotus*, then *Echinothrix* (Figs B**–**E in S1 File). Diadematidae sea urchins were the main contributors to bioerosion (Fig J in S1 File), in part because of their large test size relative to *Echinometra* (Fig N in S1 File) — since there is a positive, non-linear relationship between test size and rates of bioerosion — and also in part because the genera *Diadema* and *Echinothrix* were combined in estimates of bioerosion following [51]. The average bioerosion across all depths combined was ~4.8 kg CaCO_3_ m^-2^ y^-1^, with the lowest rate of ~2.4 kg CaCO_3_ m^-2^ y^-1^ at 17 m, and the highest rate of ~8.3 kg CaCO_3_ m^-2^ y^-1^ at 3 m depth (Table 1).

**Fig 4:**
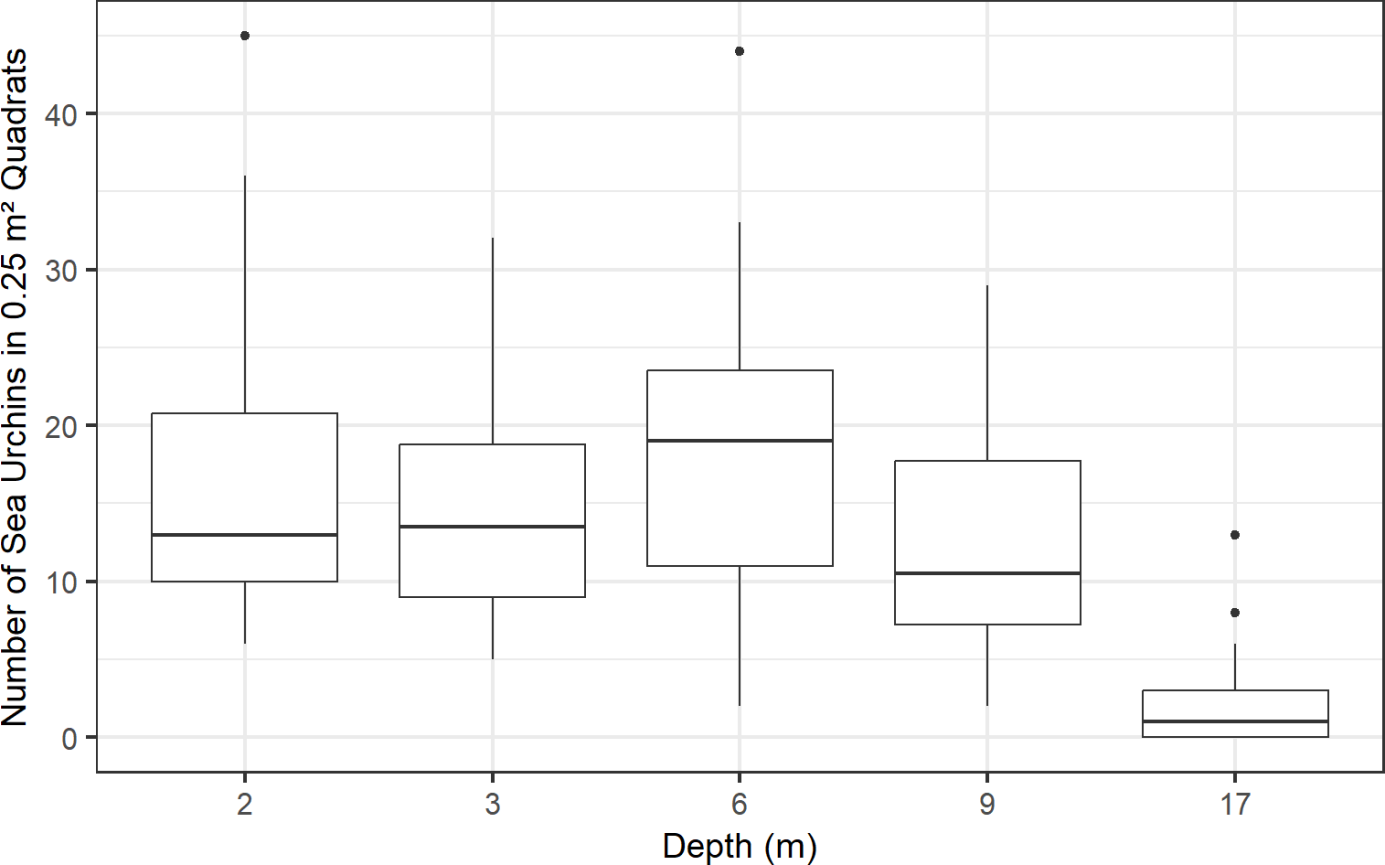
Number of all sea-urchin species surveyed in 150 x 0.25 m² quadrats at Sites 1 and 2 across depths (2–17 m) in Hōnaunau Bay, Hawai'i Island, 2023. The thick horizontal lines indicate medians, the boxes indicate the first and third quartiles, the whiskers indicate the range of the data, and the points indicate outliers. A linear mixed-effects model and Tukey post-hoc test showed that the number of sea urchins in quadrats was significantly lower (p ≤ 0.01) at 17 m than at all other depths, and at 9 m versus 6 m.

A linear mixed-effects model and Tukey post-hoc test showed that the number of sea urchins in quadrats was significantly lower (p ≤ 0.01) at 17 m than at all other depths, and at 9 m versus 6 m (Fig 4). There was a slight increase of the non-eroding species, *Tripneustes gratilla*, with depth (Table 2). The overall average number of sea urchins surveyed in 0.25 m² quadrats, across all depths combined, was ~12.7 for all species, ~1.7 excluding *Echinometra*, and ~11 for *Echinometra* only (Table 2). Scaling-up measurements from the survey size of 0.25 m² to 1 m², these values equate to an average density of ~51 individuals/m^2^ for all species, ~7 individuals/m^2^ excluding *Echinometra*, and ~44 individuals/m^2^ for *Echinometra* only. Notably, one quadrat at 2 m at Site 1 supported 45 individuals/0.25 m² (~180 individuals/m^2^) of sea urchins (Fig 4), comprised mainly of *Echinometra mathaei* (Figs C**–**E in S1 File).

**Table 2:** Average number of sea urchins by species surveyed in 150 x 0.25 m² quadrats at Sites 1 and 2 across depths (2–17 m) in Hōnaunau Bay, Hawai'i Island, 2023.

Depth (m)	Diadematidae	*Echinometra*	Other Eroding Sea Urchins	*Tripneustes gratilla*
2	0.87 ± 0.22	15.13 ± 1.65	0.30 ± 0.10	0.10 ± 0.10
3	1.17 ± 0.28	12.17 ± 1.23	0.90 ± 0.19	0.10 ± 0.06
6	0.40 ± 0.12	15.57 ± 1.52	2.07 ± 0.39	0.20 ± 0.09
9	0.17 ± 0.08	10.37 ± 1.01	1.07 ± 0.41	0.50 ± 0.16
17	0.33 ± 0.10	1.73 ± 0.54	0.27 ± 0.11	0.23 ± 0.09

Diadematidae included species in the genera *Diadema* and *Echinothrix*, *Echinometra* included species only in the genus *Echinometra*, and Other Eroding Sea Urchins included all other sea-urchin species surveyed in this study, except for the non-eroding species *Tripneustes gratilla*. Mean values across depths ± standard error (SE) are displayed. The overall mean ± SE number of sea urchins surveyed in 150 x 0.25 m² quadrats, across all depths combined, was 12.73 ± 0.75 for all species, 1.73 ± 0.16 excluding *Echinometra*, and 10.99 ± 0.69 for *Echinometra* only. Scaling-up measurements from the survey size of 0.25 m² to 1 m², these values equated to an average density of 51 individuals/m^2^ for all species, 7 individuals/m^2^ excluding *Echinometra*, and 44 individuals/m^2^ for *Echinometra* only.

### Coral-cover thresholds

**Fig 5:**
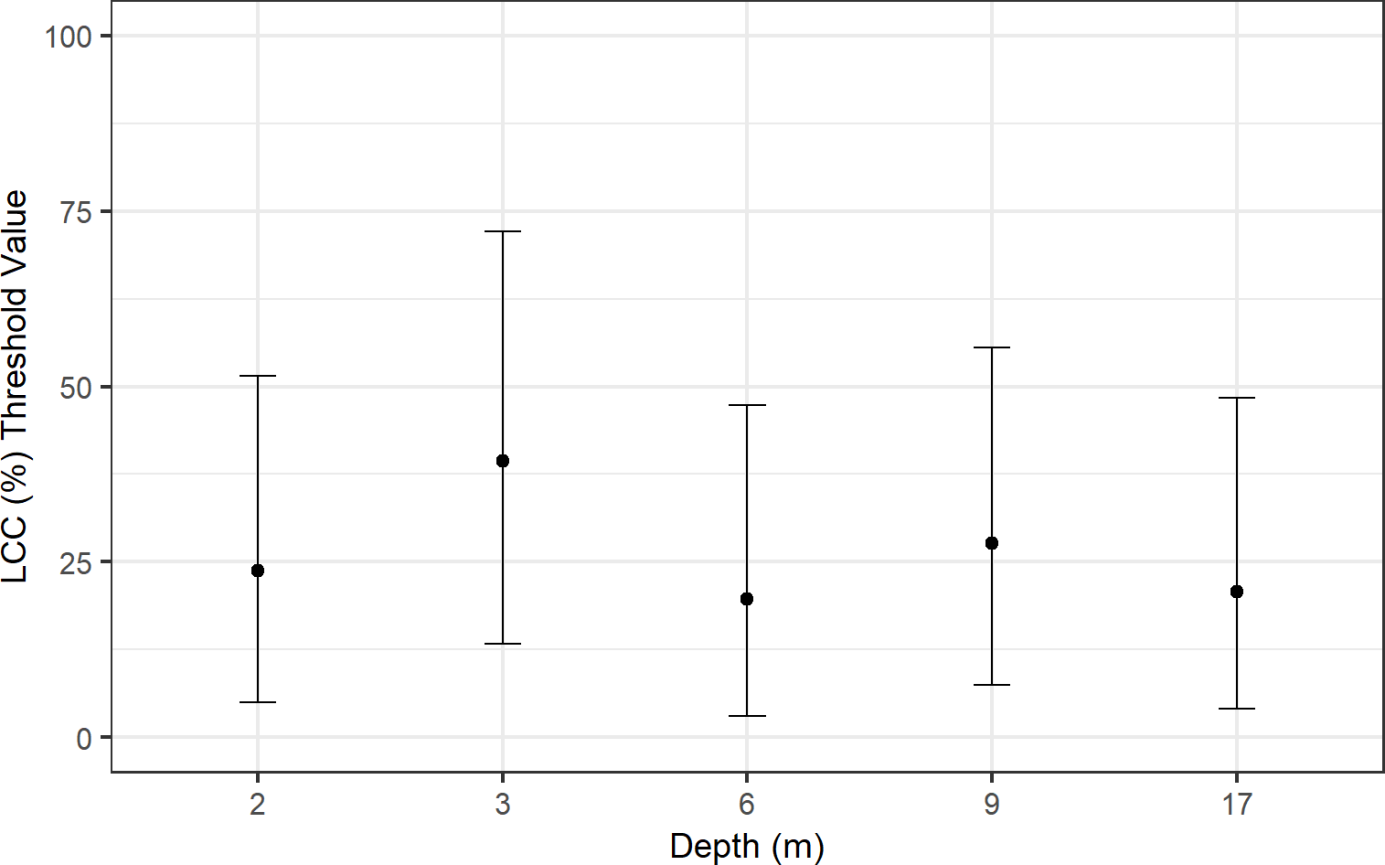
Percent threshold of live coral cover (LCC) needed to maintain positive net accretion at Sites 1 and 2 across depths (2–17 m) in Hōnaunau Bay, Hawai'i Island, 2023. The points indicate posterior means and the vertical bars indicate the 95% credible intervals.

Using an additive mixed-effects model in a Bayesian framework, this study found that the reef in Hōnaunau Bay needs to support ~26% live coral cover (LCC) to maintain positive net carbonate production across depths. Live coral cover ranged from ~19.8% needed at 6 m to ~39.4% needed at 3 m (where bioerosion was highest), to maintain positive net carbonate production (Fig 5 and Table 3). The average percent of live coral cover across all depths combined was ~28% from field surveys (Table 3), and a linear mixed-effects model and Tukey post-hoc test showed no significant differences in the percent of live coral cover surveyed among depths (Fig O in S1 File).

**Table 3:** Percent threshold of live coral cover (LCC), with 95% credible intervals (CI), needed to maintain positive net accretion, and percent of LCC surveyed in 150 x 2 m transects at Sites 1 and 2 across depths (2–17 m) in Hōnaunau Bay, Hawai'i Island, 2023.

Depth (m)	Threshold of % LCC needed to maintain positive net accretion	Lower 95% CI (% LCC)	Upper 95% CI (% LCC)	% LCC surveyed
2	23.70	5.05	51.45	23.23 ± 2.77
3	39.39	13.30	72.07	28.16 ± 3.23
6	19.76	2.99	47.32	31.16 ± 4.06
9	27.65	7.47	55.57	34.65 ± 3.47
17	20.79	4.02	48.35	25.11 ± 4.43

Threshold values for percent of LCC and credible intervals are displayed in Fig 5. The average percent threshold of LCC across all depths combined was 26.26%. For the percent of LCC surveyed, mean values across depths ± standard error are displayed, and the average across depths was 28.46 ± 1.64%.

### Scaling-up field estimates

The percent substrate available for algal growth (i.e., carbonate, rubble, coralline algae, and *Halimeda*) was highly collinear with percent live coral cover in the field estimates. Therefore, the former was removed in further analyses, and remaining field variables, which matched available airborne data from the GAO, included percent live coral cover, depth, and rugosity.

**Fig 6:**
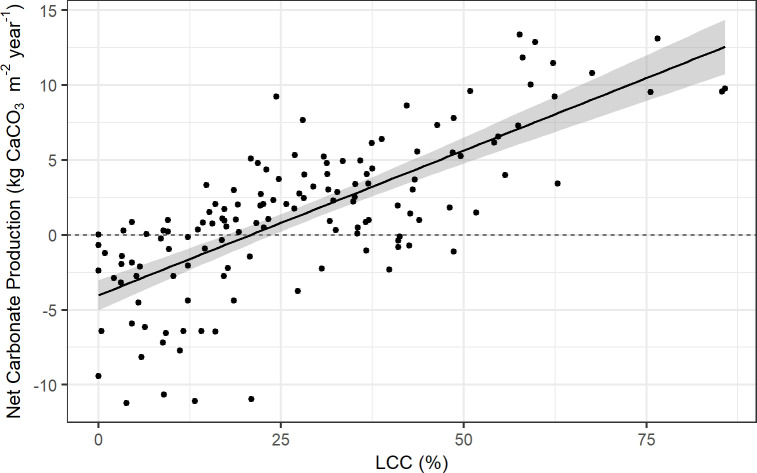
Relationship between percent live coral cover (LCC) and net carbonate production rates of the reef (kg CaCO_3_m^-2^yr^-1^) at Sites 1 and 2 across depths (2–17 m) in Hōnaunau Bay, Hawai'i Island, 2023. The regression is represented by the linear equation Net Carbonate Production = 0.19321 * (Percent LCC) – 4.01792 (p < 0.001, R^2^ = 0.54). The shading around the line indicates the 95% confidence interval. Note that outliers were removed from the regression.

**Fig 7:**
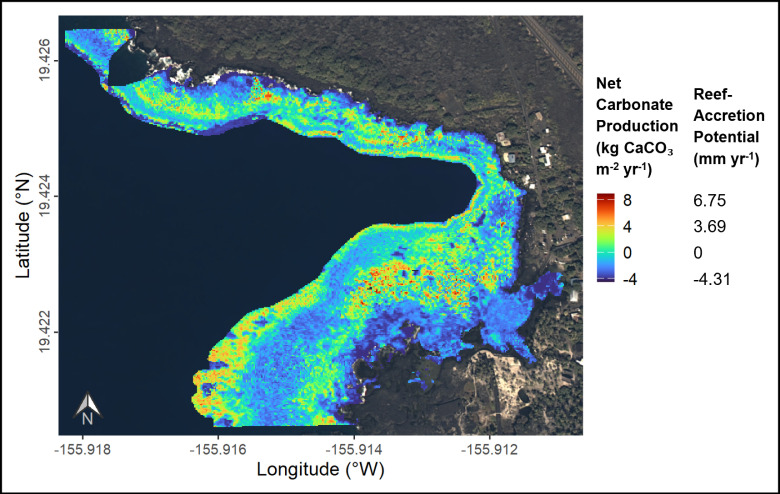
Predicted net carbonate production rates of the reef (kg CaCO_3_ m^-2^ yr^-1^) at Sites 1 and 2 across depths (2–17 m) in Hōnaunau Bay, Hawai'i Island, 2023. Net carbonate production was predicted by applying the linear regression from Fig 6 to the 2023 map of live coral cover derived from airborne imaging spectroscopy data collected by the Global Airborne Observatory [25]. Net carbonate production translates to reef-accretion potential (mm yr^-1^) using Equation (21). Background image: USGS NAIP Plus from The National Map.

A partial least squares regression (PLSR), using k-fold cross-validation, was run using all field data on the three variables described above, and revealed that variance of net carbonate production was explained through one PLS component. A standard least squares linear regression found that variance of net carbonate production was sufficiently captured from live coral cover alone, as shown by the lowest root mean square error (RMSE) (Table 4). Similarly, the *R*² values illustrate that the relationship was equally strong to predict net carbonate production from live coral cover alone, as when rugosity and depth were included (Table 4). Therefore, the linear model using all field estimates of live coral cover, excluding outliers (Fig 6), was applied to the GAO data of live coral cover to predict net carbonate production in Hōnaunau Bay to a 2 m spatial resolution (Fig 7).

**Table 4:** Model results from standard least squares linear regressions run using different field predictors of net carbonate production at Sites 1 and 2 across depths (2–17 m) in Hōnaunau Bay, Hawai'i Island, 2023.

Model	RMSE	*R*^2^ Training	*R*^2^ Testing	*R*^2^ Model
LCC + Rugosity + Depth	3.09	0.58	0.44	0.55
LCC + Rugosity	3.08	0.58	0.45	0.55
LCC	3.05	0.56	0.46	0.54

Outliers of net carbonate production were excluded prior to model runs and the data were split 70:30% for Training:Testing. RMSE is the root mean square error between actual and predicted values, LCC is percent live coral cover, and depth is meters below mean-sea level.

## Discussion

### Carbonate production

Net carbonate production was found to be, on average, ~2.4 kg CaCO_3_ m^-2^ y^-1^ at 6 m, ~1.0 kg CaCO_3_ m^-2^ y^-1^ at greater depths, ~0.2 kg CaCO_3_ m^-2^ y^-1^ at 2 m, and net negative at 3 m. Although no significant differences were observed in net carbonate production among depths, significant variations among depths were apparent in gross carbonate production and bioerosion. For example, gross carbonate production was mainly dependent on the abundance of *Porites* corals — the dominant genus surveyed in this study and found in Hawaiʻi overall [67] — with the highest production rates of ~7 kg CaCO_3_ m^-2^ y^-1^ recorded at 6 m. Highest gross production at this depth is explained by the relatively low coral mortality at 6 m during the last marine heatwave that impacted the Hawaiian Islands in 2019 [62], and favorable light conditions that make 6 m the optimal depth for *Porites* growth in Hawaiʻi [45]. To the authors’ knowledge, only one other published study has assessed carbonate production on Hawaiʻi Island, which measured gross production in the southwest and reported a maximum reef-accretion potential of ~15 kg CaCO_3_ m^-2^ y^-1^ [67]. Considering that gross production can approximate net production in optimal environments in Hawaiʻi [67], the present study’s findings suggest the reef in Hōnaunau Bay is functioning suboptimally.

Rates of net carbonate production in this study are substantially lower than rates reported in the western Pacific using similar census-based estimates [21], and are comparable to low rates in shallow reefs of the tropical western Atlantic and Indian Oceans [68], and Kailua Bay, Oʻahu [48]. In the present study, net carbonate production was also low at depth — even though there was low bioerosion at depth — because of reduced growth rates of corals associated with diminishing light. Additionally, net carbonate production was low on the shallow reef because of high rates of bioerosion.

### Bioerosion

One of the most concerning outcomes of this study was the extremely high densities of sea urchins in Hōnaunau Bay, causing high rates of bioerosion and suppressing gross carbonate production. Average bioerosion across all depths combined was ~4.8 kg CaCO_3_ m^-2^ y^-1^, and the highest bioerosion rates of ~8.3 kg CaCO_3_ m^-2^ y^-1^ were recorded at 3 m. The average density of sea urchins surveyed across all depths combined was 51 individuals/m^2^ for all species, with one quadrat at 2 m supporting ~180 individuals/m^2^. Bioerosion in the Main Hawaiian Islands is known to be higher than in the Northwestern Hawaiian Islands [69], but the densities of sea urchins recorded in this study are similar to, or higher than, extreme rates of bioerosion found in Panama [70,71], the Caribbean (pre the *Diadema* die-off of 1982–83) [72], Zanzibar [73], and Kenya [74]. For example, [70] reported average increases of *Diadema* from 3 to 80 individuals/m^2^ on reefs in Pacific Panama, with total bioerosion of 10–20 kg CaCO_3_ m^-2^ y^-1^, after the 1982–83 El Niño killed a large proportion of corals through thermal stress. Similarly, a decline in coral cover in Pacific Panama, from 2016 to 2018, was associated with average increases of *Diadema* from 0.4 to 4.9 individuals/m^2^ (in the Gulf of Chiriquí) with average bioerosion of ~8 kg CaCO_3_ m^-2^ y^-1^ in 2018 [71]. In the Caribbean, before the *Diadema* die-off of 1982–83 caused densities to be < 1 individual/m^2^, densities were between 1–14 individuals/m^2^ [72]. Indeed, the present study in Hōnaunau Bay in 2023 recorded among the highest densities of sea urchins on coral reefs globally.

Free sea-urchin species (including Diadematidae, *Heterocentrotus mammillatus*, and *Tripneustes gratilla*) have increased in density over the past two decades (1999–2019) in West Hawaiʻi, from 0.15 to ~1 sea urchin per m^2^ [75]. Historical data on the densities of rock-boring sea urchins (*Echinometra*) in west Hawaiʻi are sparse, although surveys from 1968 [76] also suggest that sea-urchin densities were considerably lower than today. For example, across the depth gradient of Hōnaunau Bay in 1968, there were an average of 1–2 sea-urchin individuals/m^2^ excluding *Echinometra* [76], whereas in 2023 there were ~7 individuals/m^2^. In the nearby Kaʻawaloa Cove at 2 m depth in 1968, there were ~10 *Echinometra* individuals/m^2^ [76], and in 2023 there were ~60 individuals/m^2^ in Hōnaunau Bay. Previously, there was a decrease in overall sea-urchin density and increase of *Tripneustes gratilla* with depth [76], which is similar to results in the present study. Also similar to the present study, the most abundant sea urchin was *Heterocentrotus*, followed by *Echinothrix* in the past when excluding *Echinometra* [76]. These comparisons suggest there has been a similar species prevalence and depth distribution of sea urchins across time, although the densities, particularly of *Echinometra*, are currently considerably higher than they were historically.

Fishing pressure, especially the lack of carnivorous fish species, is potentially one of the strongest drivers of high *Echinometra mathaei* sea-urchin densities [16–18]. For example, in Kenya, [74] found that sea-urchin densities were two orders of magnitude higher in unprotected (fished) reefs than in protected (unfished) reefs from *E. mathaei* dominance. In Zanzibar, [73] reported around 2 sea-urchin individuals/m^2^ with no *Echinometra* present on protected reefs, whereas an average of 30 individuals/m^2^ were present on fished reefs. Several studies have shown that triggerfishes are prominent predators of *E. mathaei* [77,78], with other fish predators of *E. mathaei* including terminal-male wrasses and emperor fishes [77]. Fish predators of sea urchins overall include those in the families Balistidae (triggerfishes), Labridae (wrasses), Lethrinidae (emperors), Sparidae (sea breams and porgies) and Diodontidae (porcupinefishes) [16,77,79–81]. There was a conspicuous lack of carnivorous fishes in Hōnaunau Bay in 2023, in addition to a lack of herbivorous fishes (e.g., parrotfishes). From 2008 to 2018, a 45% reduction in fish biomass was observed along west Hawaiʻi [82]. Marine Managed Areas (MMAs) are prevalent along the west coast — for example, Hōnaunau Bay is “no aquarium” [82] — but “no take” zones are rare and “no spear, no lay net, and no aquarium” zones are limited, which are known to increase fish biomass [82,83].

Herbivory by fishes can prevent and reverse shifts to macroalgal-dominated reef states [84,85], and increase coral recruitment [86] and resilience [83,87], with demonstrated benefits for controlling turf algae in Hawaiʻi [88]. Such benefits are reduced, however, when densities of herbivorous fishes are low, which was the case with parrotfish densities in this study. Herbivory by sea urchins can similarly prevent algal overgrowth (e.g., [89–91]). However, costs of high bioerosion to a reef’s carbonate budget far outweigh benefits of herbivory when sea-urchin populations are large. Moreover, herbivory by fishes and sea urchins is not functionally equivalent [92]. High densities of sea urchins can decrease densities of coral recruits, increase mortality of small corals (2–3 cm in diameter) [93], and are often associated with areas of low coral cover and high turf-algae cover [35,75]. Additionally, sea-urchin burrowing activities by high densities of *Echinometra* sea urchins [94], and large test sizes of Diadematidae [95], can cause significant bioerosion of the reef structure, as found in this study. Therefore, improved fisheries management is necessary in Hawaiʻi to control bioeroding sea urchins as the high densities of sea urchins are undermining the reef structure, and thus functionality.

### Coral-cover thresholds

It was found that the reef must support ~26% live coral cover to maintain positive net carbonate production across depths in Hōnaunau Bay. This threshold value varied from ~20% at 6 m to ~39% at 3 m; such variability can be expected since carbonate production and bioerosion vary within the reef-scale in Hawaiʻi [69]. The average threshold of ~26% live coral cover should be thought of as a boundary point, rather than an immutable value, around which the reef has a neutral carbonate budget. The surveyed average of live coral cover across all depths combined was ~28%, which is close to the threshold of ~26%, suggesting the reef in Hōnaunau Bay is at, or close to, a budget neutral state (i.e., accretionary ‘stasis’ [13]).

Surveys in the Caribbean suggest that reefs need to support around 10% live coral cover to maintain positive net production in the region [96]. However, these surveys coincided with a time in history when sea-urchin densities, and thus bioerosion, were low, so a lower percent cover of live coral could potentially sustain positive reef accretion in the Caribbean. If bioerosion rates remain high in Hōnaunau Bay, live coral cover will need to be greater than ~26% to maintain positive carbonate budgets (i.e., to support reef growth). Additionally, as rates of sea-level rise increase in the future, live coral cover will need to be considerably higher than 26% to keep pace with sea-level rise and to mitigate coastal flooding [68,97]. Threshold values of live coral cover needed for positive reef accretion should be taken into future consideration for Hawaiʻi, especially when using remote sensing to determine outplant locations [98–100].

### Sea-level rise

At 3.55 mm y^-1^ (in 2024), Hawaiʻi Island has the highest rate of sea-level rise across the Hawaiian-Island chain [101] and has one of the highest rates in the central Pacific Ocean [102]. These projections are expected to increase under different climate scenarios [103,104], so understanding the potential of a reef to keep pace with rates of sea-level rise is critical. Furthermore, in the context of reef restoration, one major goal is to support vertical reef accretion, which is crucial for tracking sea-level rise and providing coastal protection [105]. The low rates of carbonate production in this study suggest that the reefs of Hōnaunau Bay, and most likely the rest of the western reefs of Hawaiʻi Island, have little capacity to keep up with the rates of sea-level rise. For example, the vertical-accretion capacity of the reef at 6 m in Hōnaunau Bay was estimated at 2.3 mm y^-1^, whereas the current rate of sea-level rise (in 2024) is approximately 3.55 mm y^-1^. Reef accretion at other depths did not exceed an average of 1.2 mm y^-1^, whereas reef accretion at 3 m was negative, at -2.2 mm y^-1^. The vertical-accretion rates found in Hōnaunau Bay are similar to the growth capacity of reefs in the Gulf of Chiriquí [71] and elsewhere in the tropical western Atlantic and Indian Oceans [68], which are unlikely to keep pace with projected increases in sea-level rise. In addition to diminished reef-accretion capacity, low carbonate production leads to reductions of other geo-ecological functions — including sediment generation and habitat complexity [2]. These changes undermine essential ecosystem goods and services that rely upon structural maintenance and growth of the reef framework into the future [2].

### Scaling-up field estimates

The most transformative aspect of this research was applying field data to scale-up estimates of net carbonate production using remote-sensing data collected via airborne imaging spectroscopy. The best-fit model for scaling-up estimates was achieved using a 2-m resolution map of live coral cover [25], resulting in a low root mean square error between training and testing datasets. The *R*^2^ value of 0.54 is considered very reasonable for capturing ecological relationships [106].

However, coral cover is not the sole driver of community calcification on reefs. Other metrics such as reef complexity, which is strongly related to fish and invertebrate diversities, and depth, also influence structural complexity of reefs [2]. Indeed, studies have shown that live coral cover and rugosity correlate with fish biomass [39,107,108], and depth and rugosity contribute to fish-assemblage structure [41]. Fine-scale rugosity is driven by water depth [59], but interestingly, neither rugosity nor depth significantly contributed to predictions of net carbonate production in the present study, where estimates could be scaled-up solely with live coral cover. This is likely due to the variability of net carbonate production across depths and that live coral cover is a main component of the carbonate-budget model. Elsewhere in the main Hawaiian Islands, carbonate production is correlated with macroalgae cover [69], which was found to be highly collinear with coral cover in the present study. Applying estimates of net carbonate production from the scaling model presented here to a broader spatial scale along the west coast of Hawaiʻi Island would benefit from similar census-based sampling methods for field validation.

### Study limitations

Net carbonate production rates in this study are likely a maximum estimate of the true realized values because carbonate-budget models do not account for impacts of ocean acidification or physical erosion [68,109]. Future work could benefit from more species-specific growth rates and density estimates of corals across depths, particularly if running forecasts, since carbonate production is variable with changes in habitat [69,109] and pH [110,111]. Applying the model in the present study to scale-up estimates of net carbonate production in other reef locations in the Hawaiian archipelago and around the world will need further assessments of carbonate budgets and ground truthing.

## Conclusions

This study provided a first field estimate of biological carbonate budgets across depths on Hawaiʻi Island, in Hōnaunau Bay, and used high-resolution benthic-cover data, derived from airborne imaging spectroscopy, to scale-up estimates. The scaling model with the lowest root mean square error was achieved using a 2-m resolution map of live coral cover. Average rates of net carbonate production at each depth ranged from net negative production to ~2.4 kg CaCO_3_ m^-2^ y^-1^, with an average of ~0.5 kg CaCO_3_ m^-2^ y^-1^ across the 2–17 m depth gradient. Sea-urchin densities were among the highest recorded globally, suppressing reef-accretion potential in the shallow reef. These results showcase the need for improved fisheries management in Hōnaunau Bay to enhance carnivorous-fish abundances, thereby helping to reduce population densities of bioeroding sea urchins and improve reef-growth capacity. On average, Hōnaunau Bay is accreting at a low rate of 0.5 mm y^-1^ across depths, threatening the reef’s ability to keep pace with contemporary (3.55 mm y^-1^ in west Hawaiʻi in 2024) and future sea-level rise. Critically, a threshold of ~26% live coral cover is currently needed to maintain positive net production across depths, indicating that greater than ~26% live coral cover is needed to support positive reef growth. This study lays the foundation to enhance monitoring of coral reef carbonate production over increased temporal and spatial scales with airborne imaging spectroscopy to help determine where reefs are potentially keeping up with local and global anthropogenic stressors. Furthermore, this study highlights the need for management decisions in west Hawaiʻi that enhance resilient carbonate budgets of coral reefs, which provide essential geo-ecological functions and shoreline protection.

## Supporting information

S1 File Supporting figures and tables. (PDF)

S1 Data Field data. Compressed file that contains the raw field data as Excel spreadsheets.
(ZIP)
