# Supplementary material for: Scaling-up coral reef carbonate production: Sea-urchin bioerosion suppresses reef growth in Hawaiʻi
Source: PLoS One. 2025 May 28;20(5):e0324197. doi: 10.1371/journal.pone.0324197 (PMC12118839; doi:10.1371/journal.pone.0324197)
Supplement: S1 File — (PDF) [file pone.0324197.s001.pdf]

S1 File. Supporting figures and tables for:

Scaling-up coral reef carbonate production: sea-urchin bioerosion suppresses reef growth in Hawai‘i

Kelly J. van Woesik[1,#a*], Jiwei Li[2,3], and Gregory P. Asner[1,3]

[1] Center for Global Discovery and Conservation Science, Arizona State University, Hilo, Hawai‘i, United States of America

[2] Center for Global Discovery and Conservation Science, Arizona State University, Tempe, Arizona, United States of America

[3] School of Ocean Futures, Arizona State University, Tempe, Arizona, United States of America

[#a] Current Address: Center for Geospatial Analytics, North Carolina State University, Raleigh, North Carolina, United States of America

* Corresponding author

Email: kvanwoes@asu.edu (KJvW)

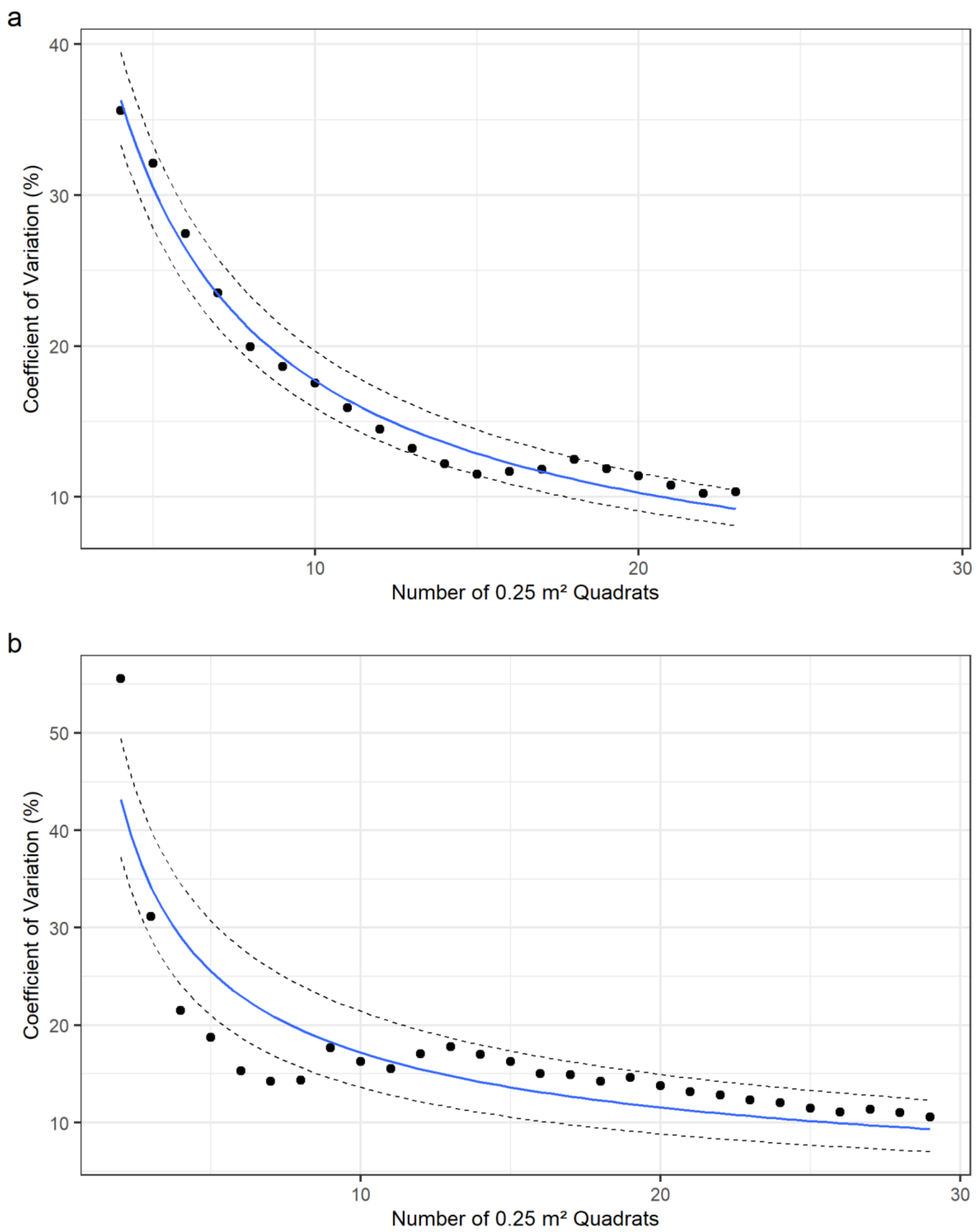

Fig A. Relationship between the coefficient of variation (CV) of mean density of the rock-boring sea urchin, *Echinometra mathaei*, and the number of 0.25 m² quadrats (n) used in field sampling, Hōnaunau Bay, Hawai‘i Island, 2023. Plots illustrate the relationship at (a) 3 m depth (n = 23) and (b) 6 m depth (n = 29). The non-linear functions follow the formula of CV ~ a * (n)^b, and level off at n = 15. Therefore, n = 15 was used in subsequent field sampling to capture the variation in *Echinometra mathaei* sea urchins, the most abundant species (Figs B-E), while minimizing field time.

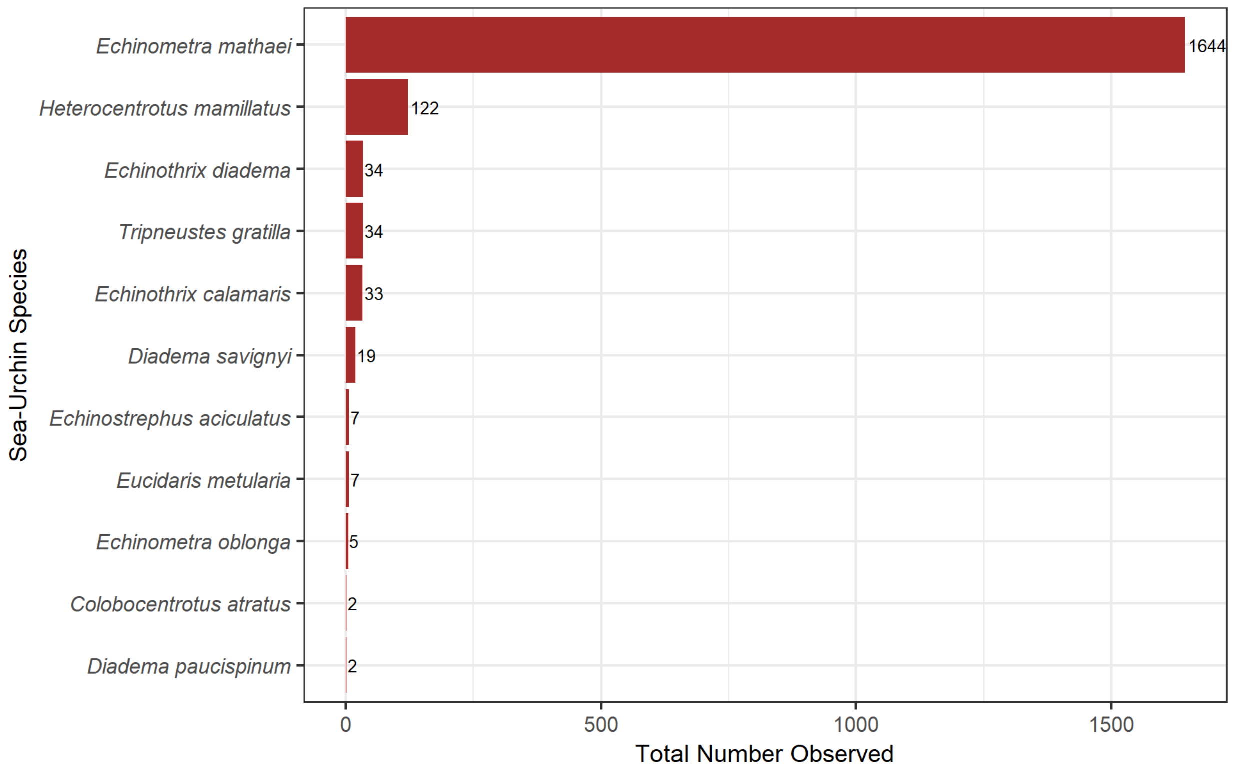

Fig B. Total number of sea urchins by species surveyed in 150 x 0.25 m² quadrats at Sites 1 and 2 across depths (2–17 m) in Hōnaunau Bay, Hawai'i Island, 2023.

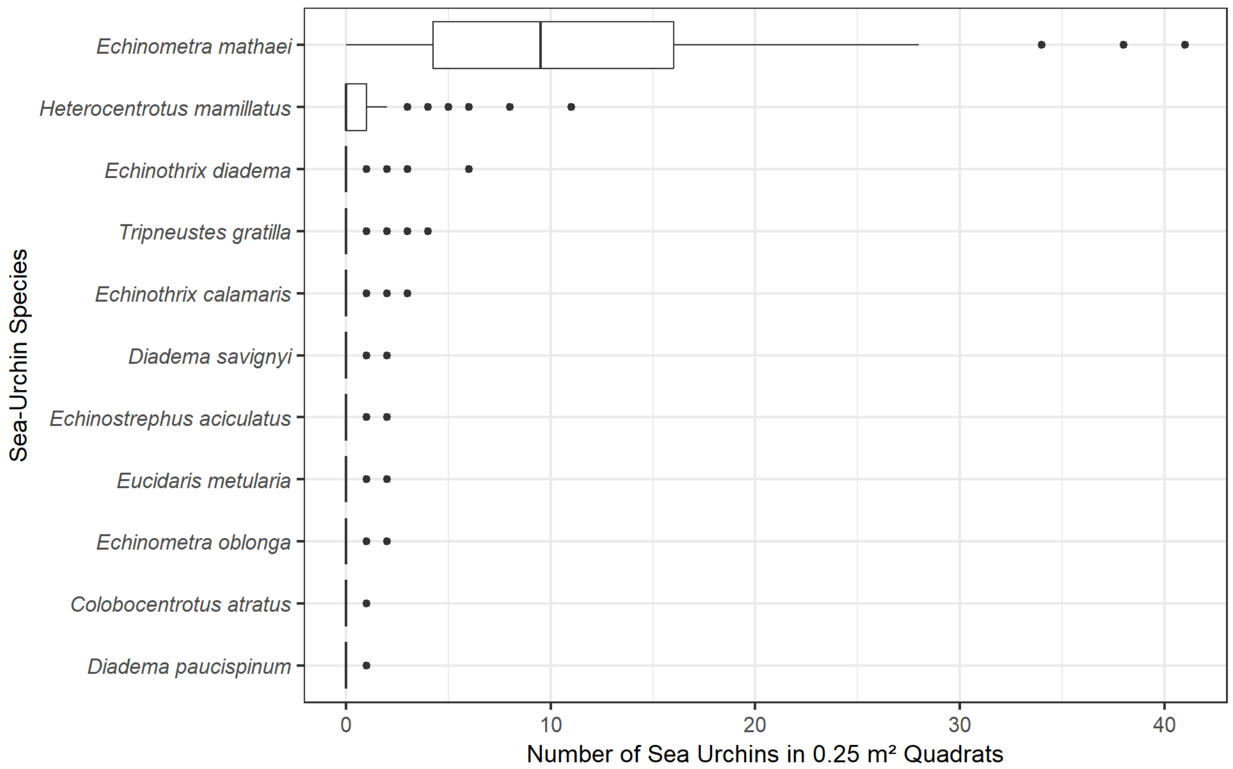

Fig C. Number of sea urchins by species surveyed in 150 x 0.25 m² quadrats at Sites 1 and 2 across depths (2–17 m) in Hōnaunau Bay, Hawai'i Island, 2023. The thick vertical lines indicate medians, the boxes indicate the first and third quartiles, the whiskers indicate the range of the data, and points indicate outliers.

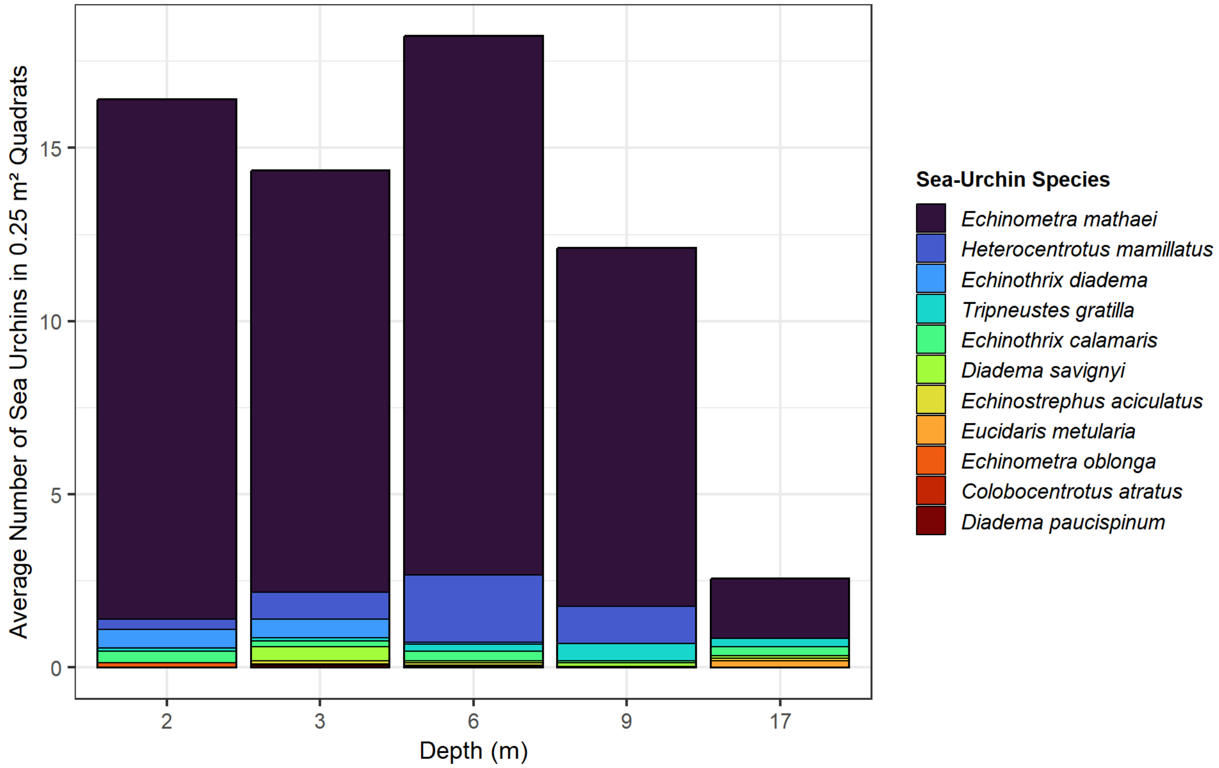

Fig D. Average number of sea urchins by species surveyed in 150 x 0.25 m² quadrats at Sites 1 and 2 across depths (2–17 m) in Hōnaunau Bay, Hawaiʻi Island, 2023.

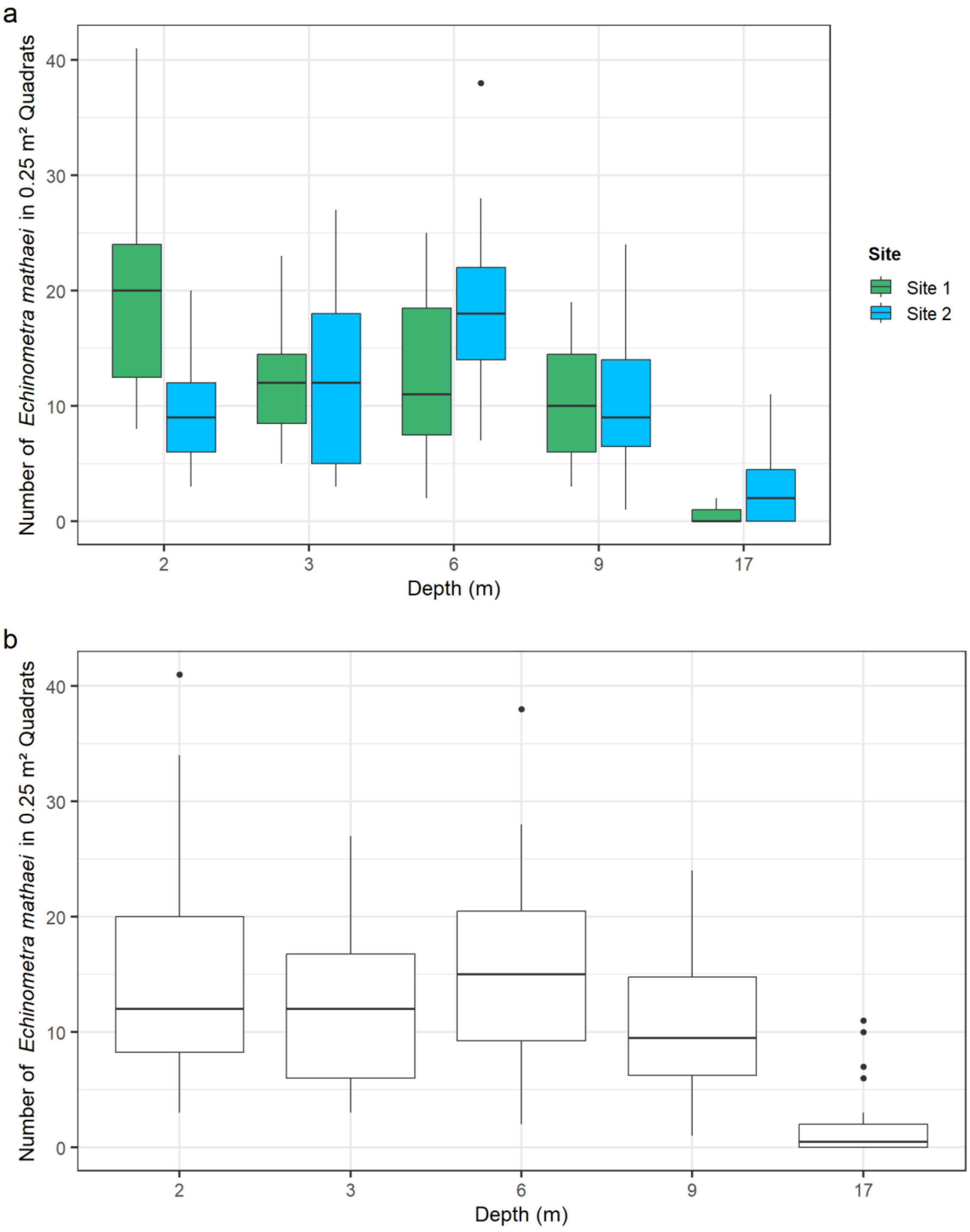

Fig E. Number of *Echinometra mathaei* sea urchins surveyed in 150 x 0.25 m² quadrats at Sites 1 and 2 across depths (2–17 m) (a) split by site and (b) with sites combined in Hōnaunau Bay, Hawai'i Island, 2023. The thick horizontal lines indicate medians, the boxes indicate the first and third quartiles, the whiskers indicate the range of the data, and points indicate outliers.

Table A. Depth adjustments applied to survey depths in Hōnaunau Bay, Hawai‘i Island, 2023 relative to Mean Sea Level (MSL).

Date (m/dd/yyyy)	SCUBA Dive	Tide Relative to MSL (m)
5/13/2023	1	-0.03
5/13/2023	2	0.02
5/13/2023	3	0.02
5/13/2023	4	-0.02
5/15/2023	1	0.05
5/15/2023	2	0.25
5/16/2023	1	0.01
5/16/2023	2	0.34
5/19/2023	1	-0.24
5/19/2023	2	0.24
5/20/2023	1	-0.25
5/20/2023	2	0.20

Tides were based on Mean Sea Level (MSL) datum from historical data at tidesandcurrents.noaa.gov, using the nearby station 1617433 (Kawaihae, HI) [1], and were extracted from the middle of the dive time. The adjustment was made so depths measured in the field could be comparable to the MSL depths recorded by the airborne data [2,3].

Table B. Adjustment coefficients for morphologies of Indo-Pacific corals.

Coral Morphology	Morphology Adjustment Coefficient
Branching	0.138
Encrusting	1
Foliose	0.2
Massive	1

The adjustment coefficients [4] were used in Equation (3) from the main text to estimate the contribution of reef-building corals to gross carbonate production.

Table C. Skeletal densities of corals used in estimates of gross carbonate production.

Family	Genus	Species	Morphology	Mean Density (g cm^{-3})	95% Confidence Interval
Acroporidae	*Montipora*	*flabellata*	Encrusting	1.207	0.346
Acroporidae	*Montipora*	*patula*	Encrusting	1.207	0.346
Acroporidae	*Montipora*	*capitata*	Encrusting	1.207	0.346
Agariciidae	*Gardineroseris*	*planulata*	Massive	1.630	0.111
Agariciidae	*Leptoseris*	*foliosa*	Foliose	1.396	0.183
Agariciidae	*Pavona*	*varians*	Encrusting	1.960	0.144
Leptastreidae	*Leptastrea*	*transversa*	Encrusting	1.630	0.317
Pocilloporidae	*Pocillopora*	*meandrina*	Branching	1.420	0.000
Poritidae	*Porites*	*compressa*	Branching	1.264	0.051
Poritidae	*Porites*	*lobata*	Massive	1.264	0.051
Poritidae	*Porites*	*monticulosa*	Massive	1.264	0.051

Densities were averaged to the genus/morphology level from published studies in the Indo-Pacific listed in [5].

Table D. Vertical growth rates of corals used in estimates of gross carbonate production.

Family	Genus	Species	Morphology	Mean Annual Extension Rate (cm yr^{-1})	95% Confidence Interval	Notes
Acroporidae	*Montipora*	*flabellata*	Encrusting	1.616	0.384	From genus/morphology average rate listed in [5] for Hawai‘i (depths not specified).
Acroporidae	*Montipora*	*patula*	Encrusting	1.616	0.384	Same as above.
Acroporidae	*Montipora*	*capitata*	Encrusting	1.616	0.384	Same as above.
Agariciidae	*Gardineroseris*	*planulata*	Massive	0.835	0.417	From genus/morphology average rate listed in [5] for the Indo-Pacific (3 mean rates from 2-3 m depth, 1 mean rate from 8 m depth).
Agariciidae	*Leptoseris*	*foliosa*	Foliose	1.968	0.659	From genus/morphology rate listed in [5] for the Indo-Pacific (depth not specified).
Agariciidae	*Pavona*	*varians*	Encrusting	1.510	0.627	From species rate listed in [5] for Hawai‘i (depth not specified).
Leptastreidae	*Leptastrea*	*transversa*	Encrusting	0.840	0.322	From genus/morphology average rate listed in [5] for the Indo-Pacific (1 mean rate from 2 m depth, 2 mean rates from 8 m depth).
Pocilloporidae	*Pocillopora*	*meandrina*	Branching	1.897	0.464	From species average rate listed in [5] for Hawai‘i (depths not specified).
Poritidae	*Porites*	*compressa*	Branching	Determined using Equations 4 & 5	-	From genus rate for Hawai‘i in [6] following the linear growth-rate Equation (4) for depths < 6 m, and the exponential growth-rate Equation (5) for depths ≥ 6 m.
Poritidae	*Porites*	*lobata*	Massive	Determined using Equations 4 & 5	-	From species rate for Hawai‘i in [6] following the linear growth-rate Equation (4) for depths < 6 m, and the exponential growth-rate Equation (5) for depths ≥ 6 m.
Poritidae	*Porites*	*monticulosa*	Massive	Determined using Equations 4 & 5	-	From genus rate for Hawai‘i in [6] following the linear growth-rate Equation (4) for depths < 6 m, and the exponential growth-rate Equation (5) for depths ≥ 6 m.

Equations listed in the table above reference those from the main text.

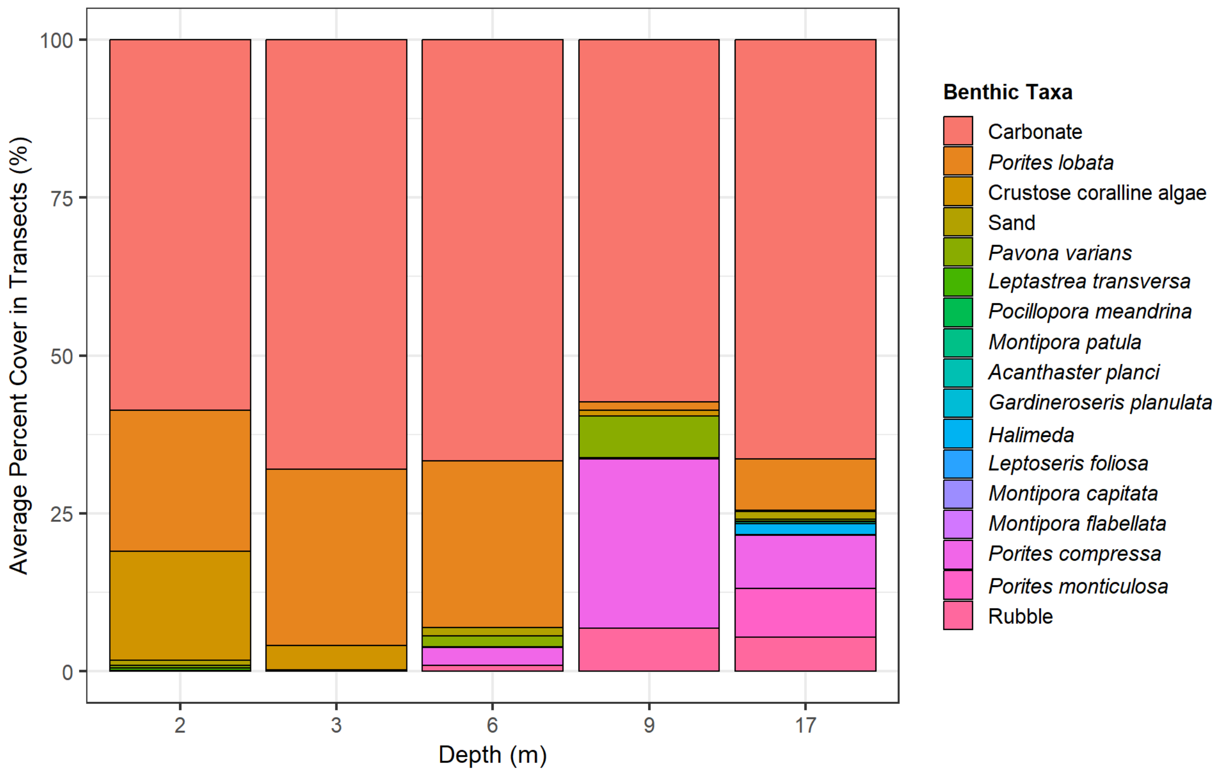

Fig F. Average percent cover of biotic and abiotic benthic components surveyed in 150 x 2 m transects at Sites 1 and 2 across depths (2–17 m) in Hōnaunau Bay, Hawai‘i Island, 2023.

Table E. Bite-rate constants of parrotfishes used in estimates of bioerosion.

Genus	Species	Bite Rate Constant	Notes
Calotomus	*carolinus*	0.244	Generic separation of *Calotomus* and *Scarus* parrotfishes is largely from dentition: *Calotomus* consume less calcium carbonate by volume and more algae material than other *Scarus* species and *Chlorurus spilurus* [7]. Therefore, a bite-rate constant value half of that for *C. spilurus* was assigned for this study.
Chlorurus	*spilurus*	0.487	Note that *Chlorurus sordidus* has been split: populations in the Red Sea and Indian Ocean are *C. sordidus* while Pacific populations are now *C. spilurus*.
Scarus	*psittacus*	0.998	
Scarus	*dubius*	0.244	Endemic to Hawaiʻi. The same bite-rate constant for *C. carolinus* was used for *S. dubius* because they feed similarly [7].
Scarus	*rubroviolaceus*	0.804	

Unless otherwise assigned and noted in the table, bite-rate constants were extracted from [4].

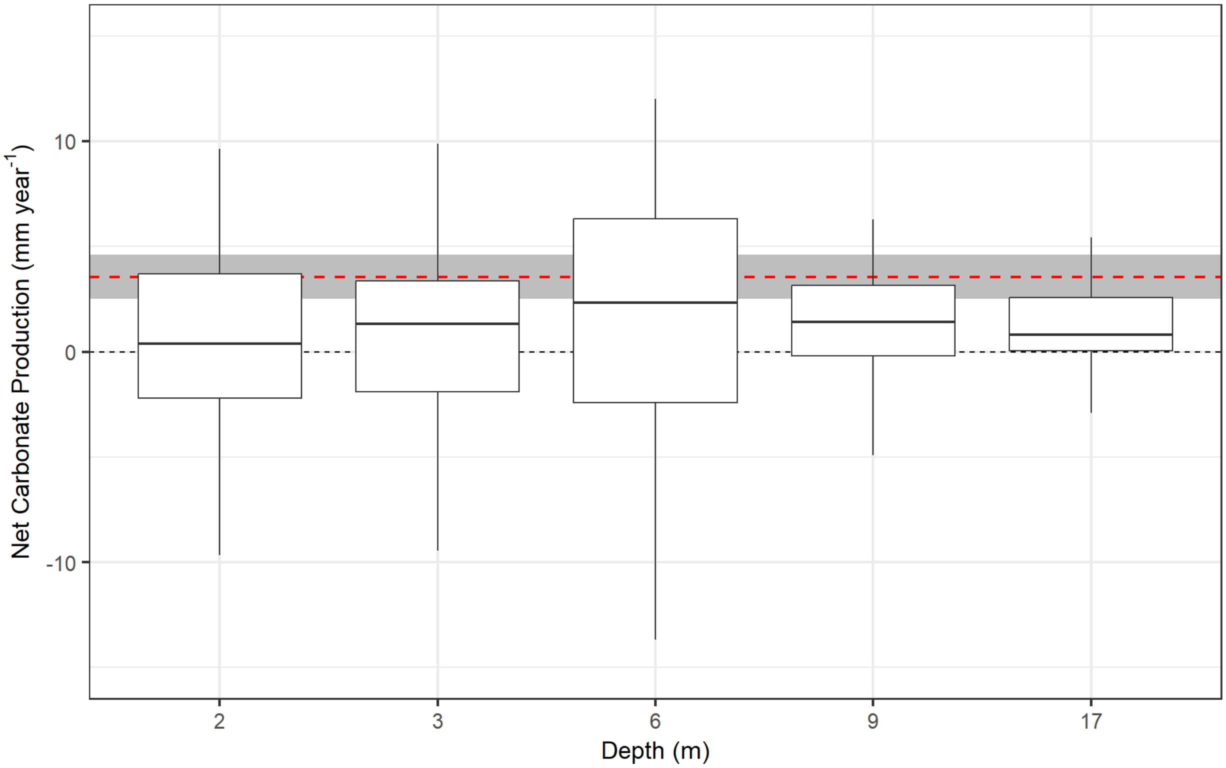

Fig G. Net vertical accretion of the reef (mm yr^{-1}) at Sites 1 and 2 across depths (2–17 m) in Hōnaunau Bay, Hawaiʻi Island, 2023. Net carbonate production translates to reef-accretion potential using Equation (21) from the main text. The thick horizontal lines indicate medians, the boxes indicate the first and third quartiles, and the whiskers indicate the range of the data. Note that outliers were removed from the display. The red horizontal dashed line is the current rate of sea-level rise (in 2024) for nearby Kawaihae, Hawaiʻi (NOAA Station 1617433), which is 3.55 mm yr^{-1} (+/- 1.05 mm yr^{-1} 95% CI in gray), based on monthly mean sea-level data from 1988 to 2023 [8].

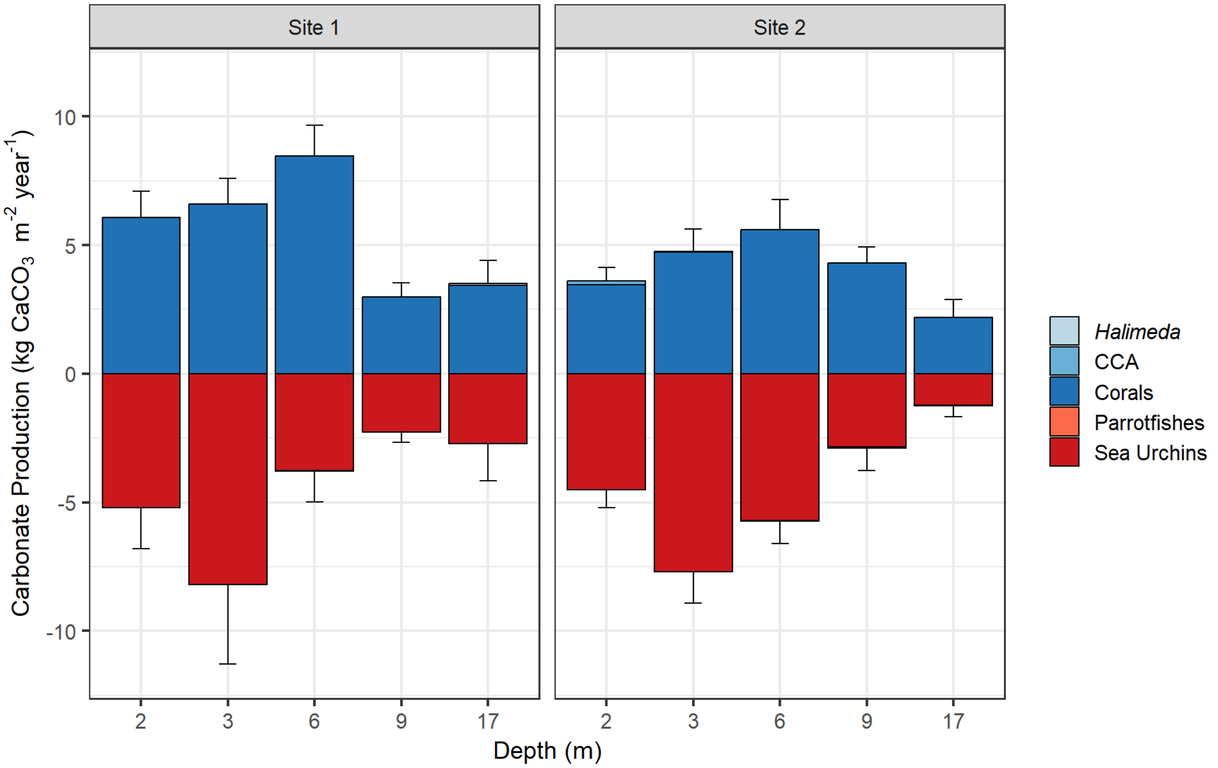

Fig H. Gross carbonate production of calcifying benthic taxa (above zero) and bioerosion by eroding group (below zero) (kg $CaCO_3$ m^{-2} yr^{-1}) at Sites 1 and 2 across depths (2–17 m) in Hōnaunau Bay, Hawai'i Island, 2023. Mean values for each group across sites and depths ± the standard error for the gross production (depicted in blue) or bioerosion (depicted in red) are displayed. Corals and sea urchins were the main contributors to gross carbonate production, whereas the contributions of *Halimeda*, crustose coralline algae (CCA), and parrotfishes were near negligible. Constants used in the carbonate-production estimates (i.e., sedimentation, macrobioerosion, and microbioerosion) are not displayed.

Table F. Coral reef carbonate budgets at Sites 1 and 2 across depths (2–17 m) in Hōnaunau Bay, Hawaiʻi Island, 2023.

Site	Depth (m)	Latitude	Longitude	Rugosity	Live Coral Cover (%)	Substrate & Algae (%)	Gross Production (kg $CaCO_3$ m^{-2} y^{-1})	Bioerosion (kg $CaCO_3$ m^{-2} y^{-1})	Net Production (kg $CaCO_3$ m^{-2} y^{-1})	Reef-Accretion Potential (mm y^{-1})
1	2	19.42238063	-155.9123718	1.25 ± 0.04	28.81 ± 4.44	69.58 ± 4.69	6.07 ± 1.03	-5.55 ± 1.61	1.05 ± 1.76	1.03 ± 1.91
1	3	19.42276884	-155.9124663	1.26 ± 0.04	32.13 ± 4.68	67.87 ± 4.68	6.60 ± 0.98	-8.56 ± 3.08	-1.44 ± 3.45	-1.48 ± 5.22
1	6	19.42277558	-155.9125681	1.47 ± 0.08	38.13 ± 5.80	59.30 ± 5.44	8.47 ± 1.20	-4.16 ± 1.22	4.85 ± 1.92	4.39 ± 1.69
1	9	19.42316929	-155.9127083	1.35 ± 0.04	38.78 ± 5.34	61.22 ± 5.34	2.98 ± 0.55	-2.60 ± 0.41	0.91 ± 0.73	0.89 ± 0.70
1	17	19.42350768	-155.9127949	1.27 ± 0.03	31.83 ± 7.64	66.14 ± 7.65	3.50 ± 0.90	-3.08 ± 1.44	0.95 ± 1.84	0.94 ± 2.13
2	2	19.42234405	-155.9133367	1.16 ± 0.02	17.66 ± 2.74	82.34 ± 2.74	3.60 ± 0.54	-4.84 ± 0.68	-0.71 ± 0.84	-0.72 ± 0.88
2	3	19.42245825	-155.9133688	1.20 ± 0.02	24.19 ± 4.36	75.69 ± 4.39	4.75 ± 0.87	-8.07 ± 1.20	-2.79 ± 1.68	-2.94 ± 2.09
2	6	19.42259985	-155.9134185	1.35 ± 0.04	24.19 ± 5.27	75.81 ± 5.27	5.59 ± 1.18	-6.16 ± 0.88	-0.04 ± 1.77	-0.04 ± 1.73
2	9	19.42327068	-155.9136699	1.29 ± 0.05	30.52 ± 4.37	69.13 ± 4.37	4.31 ± 0.61	-3.25 ± 0.89	1.59 ± 1.18	1.54 ± 1.25
2	17	19.42350759	-155.9137571	1.39 ± 0.03	18.38 ± 4.05	81.08 ± 4.01	2.19 ± 0.69	-1.70 ± 0.44	1.02 ± 0.88	1.00 ± 0.83

Gross production is the rate of carbonate production, excluding sedimentation and bioerosion rates. Bioerosion is the rate of erosion from sea urchins, parrotfishes, macrobioerosion, and microbioerosion. Net production is gross carbonate production plus sedimentation minus bioerosion rates. Net production translates to reef-accretion potential using Equation (21) from the main text. Mean values across sites and depths ± standard error are displayed.

Table G. Coral reef carbonate budgets of the 150 field replicates at Sites 1 and 2 across depths (2–17 m) in Hōnaunau Bay, Hawai‘i Island, 2023.

Site	Average Site Depth (m)	Transect/ Quadrat Replicate	Quadrat Depth (m)	Rugosity	Live Coral Cover (%)	Substrate & Algae (%)	Gross Production (kg $CaCO_3$ m^{-2} y^{-1})	Bioerosion (kg $CaCO_3$ m^{-2} y^{-1})	Net Production (kg $CaCO_3$ m^{-2} y^{-1})	Reef-Accretion Potential (mm y^{-1})
1	2	1	2.05	1.46	31.27	68.73	7.44	-3.18	4.79	4.35
1	2	2	1.85	1.27	23.92	70.98	5.00	-3.21	2.32	2.22
1	2	3	1.85	1.18	33.47	47.46	6.48	-2.07	4.94	4.46
1	2	4	1.95	1.40	51.25	48.75	11.71	-20.55	-8.30	-9.65
1	2	5	2.05	1.41	16.31	83.69	3.76	-20.03	-15.74	-20.56
1	2	6	2.05	1.41	14.59	85.41	3.46	-4.88	-0.90	-0.91
1	2	7	1.85	1.18	31.36	68.64	5.94	-2.42	4.05	3.73
1	2	8	2.15	1.18	21.61	78.39	4.16	-3.91	0.78	0.77
1	2	9	2.05	1.07	35.81	64.19	6.30	-1.87	4.95	4.48
1	2	10	1.95	1.11	8.56	91.44	1.60	-2.39	-0.25	-0.26
1	2	11	1.75	1.17	28.21	71.79	5.42	-1.95	4.01	3.70
1	2	12	1.75	1.08	18.52	81.48	3.29	-8.19	-4.37	-4.75
1	2	13	1.75	1.30	57.47	42.53	12.33	-5.56	7.30	6.26
1	2	14	2.25	1.46	59.79	40.21	14.18	-1.85	12.87	9.64
1	2	15	2.15	1.15	0.00	100.00	0.00	-1.21	-0.68	-0.69
1	3	1	3.42	1.30	17.25	82.76	3.63	-2.45	1.71	1.65
1	3	2	3.82	1.56	57.69	42.31	14.43	-1.59	13.38	9.89
1	3	3	3.12	1.38	42.18	57.82	9.32	-1.22	8.64	7.18
1	3	4	3.12	1.28	37.50	62.50	7.71	-3.83	4.42	4.04
1	3	5	3.02	1.15	43.04	56.96	7.97	-5.49	3.01	2.83
1	3	6	2.92	1.12	32.44	67.56	5.89	-46.16	-39.75	-70.53
1	3	7	2.62	1.11	67.57	32.43	12.15	-1.89	10.79	8.52
1	3	8	3.02	1.21	23.14	76.86	4.51	-19.25	-14.22	-18.15
1	3	9	3.12	1.18	2.13	97.87	0.40	-3.82	-2.89	-3.05

Site	Average Site Depth (m)	Transect/ Quadrat Replicate	Quadrat Depth (m)	Rugosity	Live Coral Cover (%)	Substrate & Algae (%)	Gross Production (kg $CaCO_3$ m^{-2} y^{-1})	Bioerosion (kg $CaCO_3$ m^{-2} y^{-1})	Net Production (kg $CaCO_3$ m^{-2} y^{-1})	Reef-Accretion Potential (mm y^{-1})
1	3	10	3.42	1.39	12.23	87.77	2.72	-7.64	-4.39	-4.76
1	3	11	3.32	1.29	48.65	51.35	10.09	-2.83	7.79	6.61
1	3	12	2.92	1.51	13.91	86.09	3.93	-19.98	-15.52	-20.22
1	3	13	2.82	1.12	32.14	67.86	6.02	-4.26	2.29	2.19
1	3	14	2.62	1.07	17.21	82.79	3.00	-2.56	0.97	0.95
1	3	15	3.02	1.27	34.90	65.10	7.16	-5.45	2.24	2.14
1	6	1	5.83	1.70	20.88	79.12	6.03	-1.48	5.08	4.58
1	6	2	5.63	1.48	30.85	69.15	7.78	-3.09	5.22	4.69
1	6	3	6.13	1.60	20.94	79.06	7.26	-18.74	-10.95	-13.28
1	6	4	6.13	1.55	11.62	88.39	3.42	-10.38	-6.43	-7.24
1	6	5	6.63	1.65	16.97	68.79	3.21	-2.66	1.08	1.06
1	6	6	5.53	1.40	32.50	67.50	4.51	-4.73	0.31	0.31
1	6	7	6.73	1.18	36.59	63.40	3.28	-2.93	0.87	0.86
1	6	8	6.13	1.35	62.23	37.78	12.76	-1.81	11.48	8.91
1	6	9	6.23	1.38	85.45	14.55	18.92	-0.24	19.21	12.02
1	6	10	6.53	2.36	24.36	71.40	10.80	-2.09	9.24	7.58
1	6	11	6.63	1.38	28.00	72.00	8.59	-1.47	7.65	6.51
1	6	12	6.93	1.38	21.82	58.18	4.92	-0.66	4.79	4.34
1	6	13	6.73	1.32	54.72	45.28	10.76	-4.72	6.57	5.73
1	6	14	5.53	1.18	48.51	51.49	9.78	-4.82	5.49	4.90
1	6	15	6.53	1.24	76.52	23.48	15.09	-2.52	13.09	9.75
1	9	1	8.05	1.52	26.88	73.11	7.06	-2.27	5.32	4.77
1	9	2	7.85	1.22	31.69	68.31	2.63	-2.23	0.93	0.91
1	9	3	8.25	1.26	73.91	26.09	2.03	-1.27	1.29	1.26
1	9	4	8.65	1.28	67.18	32.81	2.53	-1.88	1.18	1.15
1	9	5	8.45	1.23	30.61	69.39	0.81	-3.60	-2.26	-2.36
1	9	6	8.95	1.56	38.78	61.22	6.92	-1.06	6.39	5.59

Site	Average Site Depth (m)	Transect/ Quadrat Replicate	Quadrat Depth (m)	Rugosity	Live Coral Cover (%)	Substrate & Algae (%)	Gross Production (kg $CaCO_3$ m^{-2} y^{-1})	Bioerosion (kg $CaCO_3$ m^{-2} y^{-1})	Net Production (kg $CaCO_3$ m^{-2} y^{-1})	Reef-Accretion Potential (mm y^{-1})
1	9	7	8.65	1.54	16.88	83.12	1.03	-1.90	-0.34	-0.34
1	9	8	7.85	1.60	51.72	48.29	3.19	-2.25	1.47	1.43
1	9	9	8.15	1.30	48.66	51.34	4.84	-6.47	-1.10	-1.12
1	9	10	8.15	1.19	48.10	51.90	3.16	-1.87	1.82	1.76
1	9	11	8.65	1.46	55.67	44.33	4.48	-1.03	3.98	3.67
1	9	12	8.35	1.47	42.52	57.48	1.90	-3.14	-0.71	-0.72
1	9	13	7.85	1.16	42.67	57.33	3.42	-2.54	1.41	1.37
1	9	14	8.15	1.12	0.89	99.11	0.02	-1.78	-1.22	-1.25
1	9	15	7.85	1.36	5.50	94.51	0.71	-5.76	-4.52	-4.91
1	17	1	18.65	1.15	54.15	45.85	5.95	-0.31	6.17	5.43
1	17	2	17.45	1.60	19.06	80.94	2.03	-0.54	2.02	1.94
1	17	3	18.25	1.22	22.23	77.78	2.60	-0.40	2.73	2.59
1	17	4	17.35	1.15	22.61	77.39	0.48	-0.51	0.50	0.49
1	17	5	16.55	1.32	0.00	100.00	0.05	-0.55	0.03	0.03
1	17	6	16.75	1.21	9.54	83.82	1.03	-0.58	0.98	0.96
1	17	7	18.45	1.12	75.55	24.44	9.11	-0.12	9.52	7.75
1	17	8	18.15	1.26	85.77	14.23	9.32	-0.08	9.77	7.91
1	17	9	16.65	1.20	85.35	14.64	9.10	-0.08	9.55	7.77
1	17	10	16.95	1.36	10.25	89.75	1.75	-5.04	-2.76	-2.91
1	17	11	16.75	1.41	27.30	48.94	5.59	-9.87	-3.75	-4.02
1	17	12	15.95	1.37	39.78	60.21	4.18	-7.02	-2.31	-2.41
1	17	13	15.85	1.22	8.23	91.77	0.35	-20.14	-19.26	-26.48
1	17	14	15.75	1.18	3.40	96.60	0.22	-0.47	0.27	0.27
1	17	15	16.05	1.23	14.28	85.71	0.74	-0.44	0.83	0.82
2	2	1	2.10	1.12	16.00	84.00	2.75	-9.74	-6.45	-7.27
2	2	2	2.60	1.23	12.24	87.76	2.43	-3.12	-0.15	-0.15
2	2	3	2.10	1.24	35.48	64.52	7.19	-7.24	0.48	0.48

Site	Average Site Depth (m)	Transect/ Quadrat Replicate	Quadrat Depth (m)	Rugosity	Live Coral Cover (%)	Substrate & Algae (%)	Gross Production (kg $CaCO_3$ m^{-2} y^{-1})	Bioerosion (kg $CaCO_3$ m^{-2} y^{-1})	Net Production (kg $CaCO_3$ m^{-2} y^{-1})	Reef-Accretion Potential (mm y^{-1})
2	2	4	2.30	1.09	27.52	72.48	4.60	-2.38	2.75	2.60
2	2	5	1.70	1.11	22.97	77.03	5.74	-1.90	4.37	4.00
2	2	6	1.70	1.06	0.00	100.00	0.38	-3.30	-2.39	-2.50
2	2	7	1.90	1.08	9.21	90.78	1.71	-8.80	-6.56	-7.40
2	2	8	1.50	1.13	35.40	64.60	6.86	-7.31	0.08	0.08
2	2	9	1.50	1.24	17.74	82.26	3.82	-6.56	-2.21	-2.30
2	2	10	1.40	1.09	4.59	95.41	1.05	-3.42	-1.85	-1.91
2	2	11	1.70	1.28	13.62	86.38	3.28	-3.44	0.37	0.37
2	2	12	1.80	1.14	17.18	82.82	3.61	-6.90	-2.76	-2.91
2	2	13	1.70	1.18	22.55	77.45	4.36	-2.82	2.07	1.99
2	2	14	1.60	1.24	5.67	94.33	1.15	-3.80	-2.12	-2.21
2	2	15	1.60	1.20	24.69	75.31	5.01	-1.81	3.74	3.46
2	3	1	2.86	1.10	13.18	86.82	2.34	-13.94	-11.07	-13.46
2	3	2	2.96	1.17	36.91	61.37	6.93	-4.05	3.41	3.18
2	3	3	2.86	1.12	3.11	96.89	0.56	-4.27	-3.18	-3.37
2	3	4	2.86	1.27	3.14	96.86	0.65	-3.13	-1.96	-2.03
2	3	5	2.76	1.31	36.64	63.36	7.79	-9.36	-1.04	-1.06
2	3	6	3.16	1.27	43.93	56.08	9.01	-8.56	0.99	0.97
2	3	7	2.86	1.18	8.94	91.06	1.82	-13.02	-10.66	-12.88
2	3	8	2.66	1.26	0.00	100.00	0.14	-19.09	-18.42	-25.04
2	3	9	3.06	1.34	41.26	58.74	8.98	-9.61	-0.11	-0.11
2	3	10	3.46	1.18	28.09	71.91	5.27	-3.34	2.47	2.35
2	3	11	2.86	1.13	36.73	63.27	6.70	-3.17	4.06	3.74
2	3	12	3.26	1.19	5.91	94.09	1.13	-9.82	-8.15	-9.45
2	3	13	2.76	1.15	41.05	58.95	7.60	-8.50	-0.37	-0.37
2	3	14	2.36	1.23	20.73	79.27	4.19	-6.15	-1.43	-1.47
2	3	15	2.66	1.16	43.29	56.71	8.16	-5.01	3.68	3.42

Site	Average Site Depth (m)	Transect/ Quadrat Replicate	Quadrat Depth (m)	Rugosity	Live Coral Cover (%)	Substrate & Algae (%)	Gross Production (kg $CaCO_3$ m^{-2} y^{-1})	Bioerosion (kg $CaCO_3$ m^{-2} y^{-1})	Net Production (kg $CaCO_3$ m^{-2} y^{-1})	Reef-Accretion Potential (mm y^{-1})
2	6	1	5.96	1.33	32.71	67.29	7.36	-5.02	2.87	2.71
2	6	2	6.56	1.48	41.02	58.98	10.04	-11.39	-0.82	-0.83
2	6	3	6.16	1.36	58.09	41.91	13.28	-1.99	11.82	9.10
2	6	4	6.76	1.50	16.00	84.00	4.22	-2.70	2.05	1.97
2	6	5	6.26	1.12	0.44	99.56	0.10	-7.04	-6.41	-7.21
2	6	6	6.96	1.18	3.83	96.17	0.53	-12.29	-11.23	-13.69
2	6	7	6.46	1.34	5.22	94.78	1.17	-4.44	-2.75	-2.90
2	6	8	6.46	1.55	9.68	90.32	2.50	-3.98	-0.95	-0.97
2	6	9	6.16	1.59	31.45	68.55	8.41	-5.91	3.03	2.85
2	6	10	6.56	1.32	26.79	73.21	5.89	-4.68	1.74	1.68
2	6	11	6.76	1.21	14.10	85.89	3.00	-9.96	-6.43	-7.24
2	6	12	6.16	1.23	59.18	40.82	11.99	-2.51	10.01	8.06
2	6	13	6.16	1.31	4.56	95.44	1.58	-8.03	-5.92	-6.60
2	6	14	6.66	1.36	8.82	91.18	1.99	-9.71	-7.19	-8.20
2	6	15	6.36	1.40	50.90	49.10	11.86	-2.81	9.58	7.79
2	9	1	8.99	1.12	8.89	91.11	0.64	-0.89	0.28	0.28
2	9	2	9.39	1.23	25.71	74.29	2.68	-1.17	2.04	1.96
2	9	3	8.59	1.38	23.27	76.73	2.75	-2.22	1.06	1.04
2	9	4	8.19	1.30	49.61	50.38	5.93	-1.20	5.26	4.72
2	9	5	8.79	1.12	0.00	100.00	0.00	-9.94	-9.41	-11.14
2	9	6	8.79	1.15	14.78	85.22	3.93	-1.13	3.33	3.12
2	9	7	7.49	1.48	11.15	88.85	3.38	-11.64	-7.73	-8.90
2	9	8	8.29	1.09	62.85	37.16	6.15	-3.24	3.44	3.21
2	9	9	7.99	1.81	37.02	62.98	7.14	-6.67	1.00	0.98
2	9	10	9.29	1.20	35.15	64.85	3.78	-0.91	3.40	3.18
2	9	11	8.89	1.20	41.00	59.00	5.34	-3.91	1.96	1.88
2	9	12	9.39	1.32	35.10	64.90	3.24	-1.26	2.51	2.39

Site	Average Site Depth (m)	Transect/ Quadrat Replicate	Quadrat Depth (m)	Rugosity	Live Coral Cover (%)	Substrate & Algae (%)	Gross Production (kg $CaCO_3$ m^{-2} y^{-1})	Bioerosion (kg $CaCO_3$ m^{-2} y^{-1})	Net Production (kg $CaCO_3$ m^{-2} y^{-1})	Reef-Accretion Potential (mm y^{-1})
2	9	13	9.29	1.33	37.45	62.55	7.24	-1.64	6.13	5.40
2	9	14	9.99	1.23	29.39	65.30	4.32	-1.64	3.21	3.01
2	9	15	9.49	1.45	46.37	53.63	8.14	-1.33	7.34	6.29
2	17	1	18.64	1.45	6.55	89.65	0.15	-0.62	0.06	0.06
2	17	2	18.14	1.42	4.58	95.42	0.91	-0.57	0.87	0.85
2	17	3	17.74	1.26	9.49	90.51	0.19	-0.50	0.22	0.22
2	17	4	16.54	1.59	12.27	87.74	0.78	-3.37	-2.05	-2.13
2	17	5	17.84	1.26	6.34	93.65	0.49	-7.17	-6.15	-6.89
2	17	6	16.94	1.27	18.82	76.86	1.11	-0.63	1.01	0.99
2	17	7	16.84	1.47	22.18	77.82	3.53	-2.10	1.96	1.89
2	17	8	16.94	1.28	15.57	84.44	1.05	-0.83	0.75	0.73
2	17	9	15.24	1.38	15.22	84.78	2.72	-1.71	1.53	1.49
2	17	10	16.54	1.24	3.23	96.77	0.33	-2.27	-1.40	-1.44
2	17	11	15.84	1.53	18.57	81.43	3.22	-0.75	3.00	2.82
2	17	12	15.74	1.54	17.54	82.47	2.01	-1.99	0.55	0.54
2	17	13	15.54	1.18	43.65	56.36	6.04	-1.01	5.56	4.96
2	17	14	15.64	1.39	62.46	37.55	9.88	-1.18	9.23	7.57
2	17	15	16.54	1.58	19.24	80.76	0.51	-0.86	0.18	0.17

Quadrat depth is meters below mean-sea level. Gross production is the rate of carbonate production, excluding sedimentation and bioerosion rates. Bioerosion is the rate of erosion from sea urchins, parrotfishes, macrobioerosion, and microbioerosion. Net production is gross carbonate production plus sedimentation minus bioerosion rates. Net production translates to reef-accretion potential using Equation (21) from the main text. All values were estimated at the transect/quadrat level (each 0.25 m^2 quadrat was positioned centrally over a 2 m transect), except for parrotfish bioerosion, which was averaged at the site and depth level (from six 25 x 5 m belt transects at each site and depth).

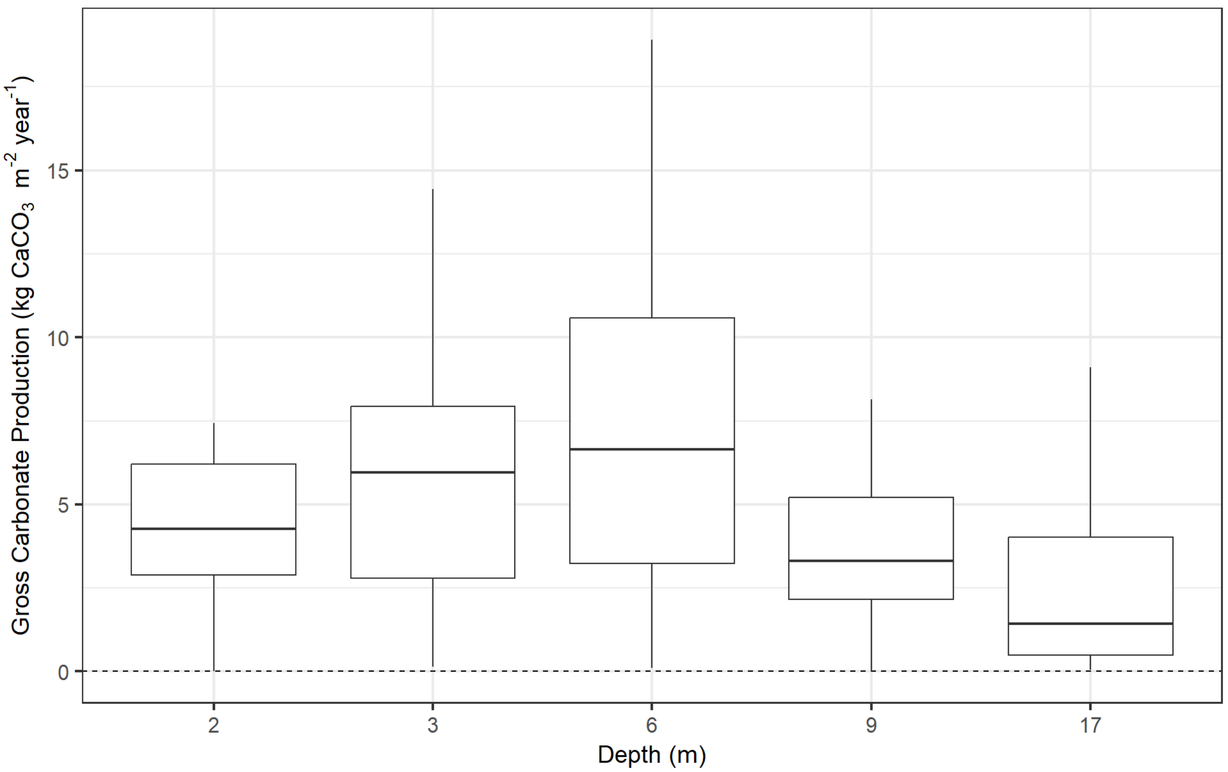

Fig I. Gross carbonate production rates of the reef (kg $CaCO_3$ m^{-2} yr^{-1}) at Sites 1 and 2 across depths (2–17 m) in Hōnaunau Bay, Hawai‘i Island, 2023. The thick horizontal lines indicate medians, the boxes indicate the first and third quartiles, and the whiskers indicate the range of the data. Note that outliers were removed from the display. A linear mixed-effects model and Tukey post-hoc test showed that gross carbonate production was significantly lower ($p \leq 0.02$) at 17 m than at 3 m and 6 m, and at 9 m versus 6 m.

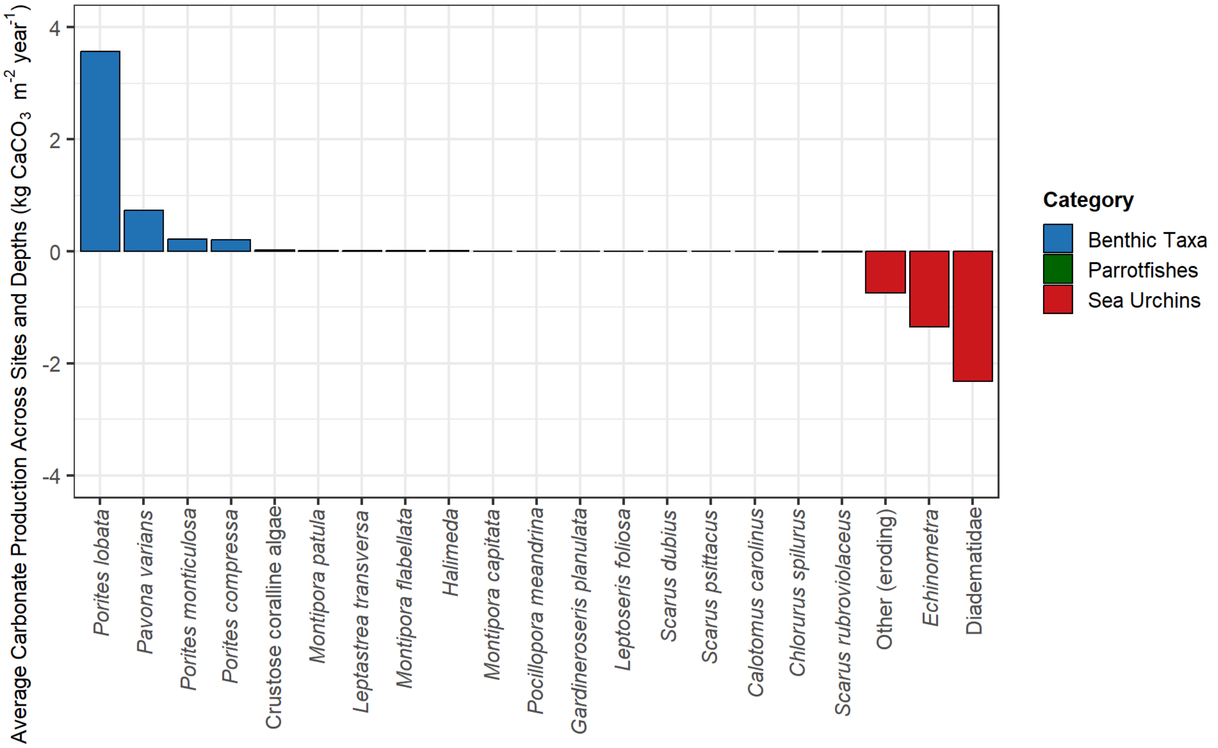

Fig J. Average gross carbonate production of calcifying benthic taxa (above zero) and bioerosion by parrotfish and sea-urchin species (below zero) (kg $CaCO_3$ m^{-2} yr^{-1}) at Sites 1 and 2 across depths (2–17 m) in Hōnaunau Bay, Hawai‘i Island, 2023. Mean values across sites and depths are displayed.

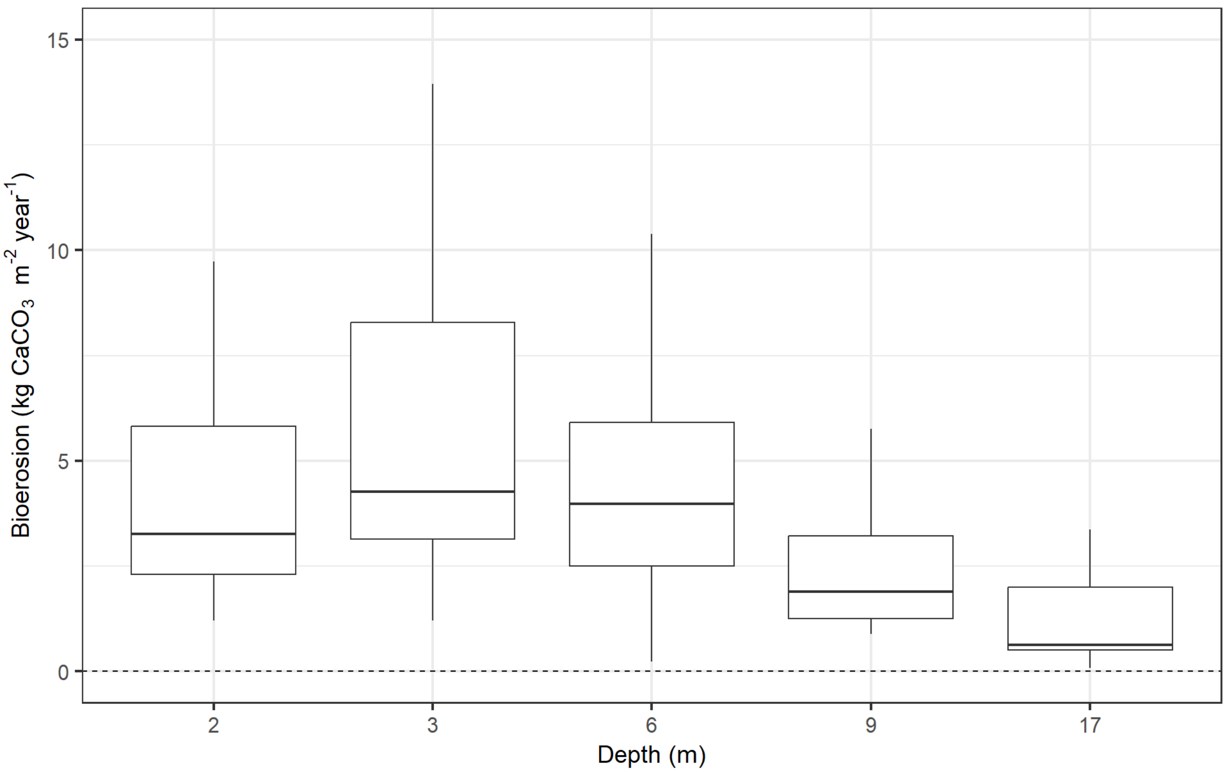

Fig K. Bioerosion rates of the reef (kg $CaCO_3$ m^{-2} yr^{-1}) at Sites 1 and 2 across depths (2–17 m) in Hōnaunau Bay, Hawai'i Island, 2023. The thick horizontal lines indicate medians, the boxes indicate the first and third quartiles, and the whiskers indicate the range of the data. Note that outliers were removed from the display. A linear mixed-effects model and Tukey post-hoc test showed that bioerosion was significantly higher ($p \leq 0.001$) at 3 m than at 9 m and 17 m.

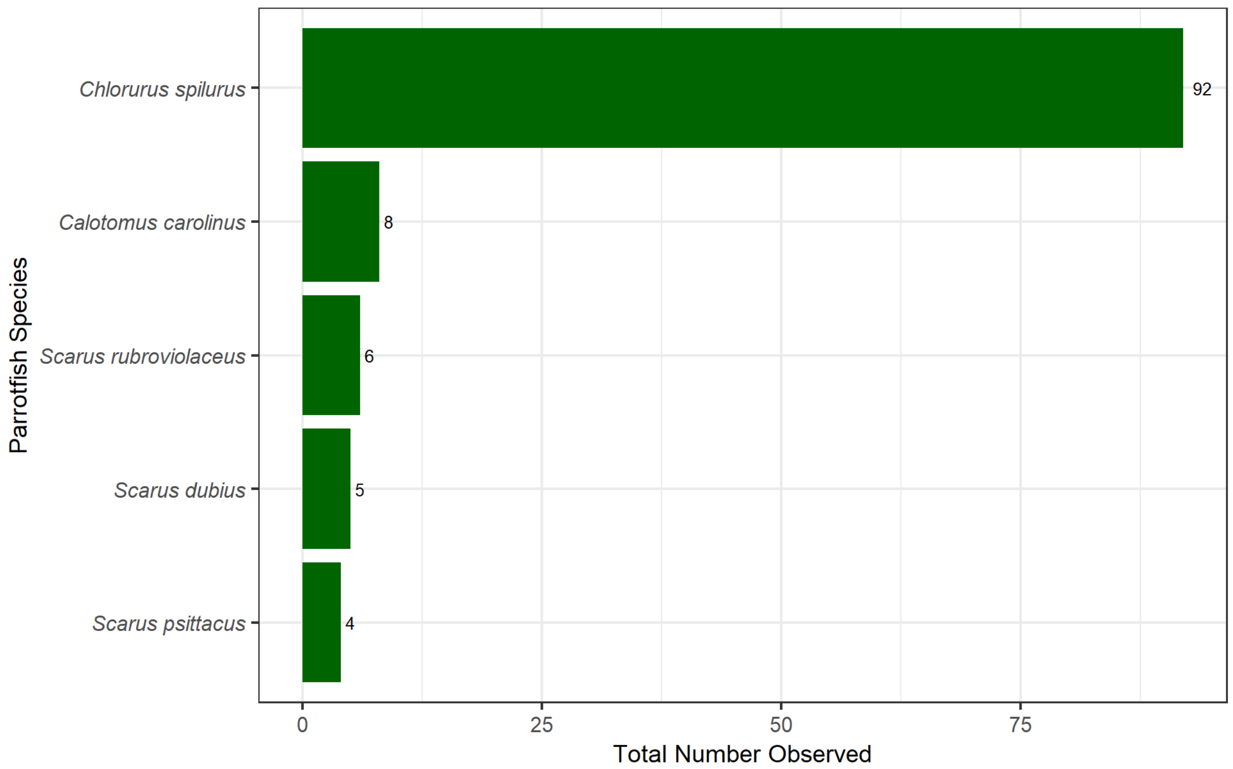

Fig L. Total number of parrotfishes by species surveyed in sixty 25 x 5 m belt transects at Sites 1 and 2 across depths (2–17 m) in Hōnaunau Bay, Hawai‘i Island, 2023.

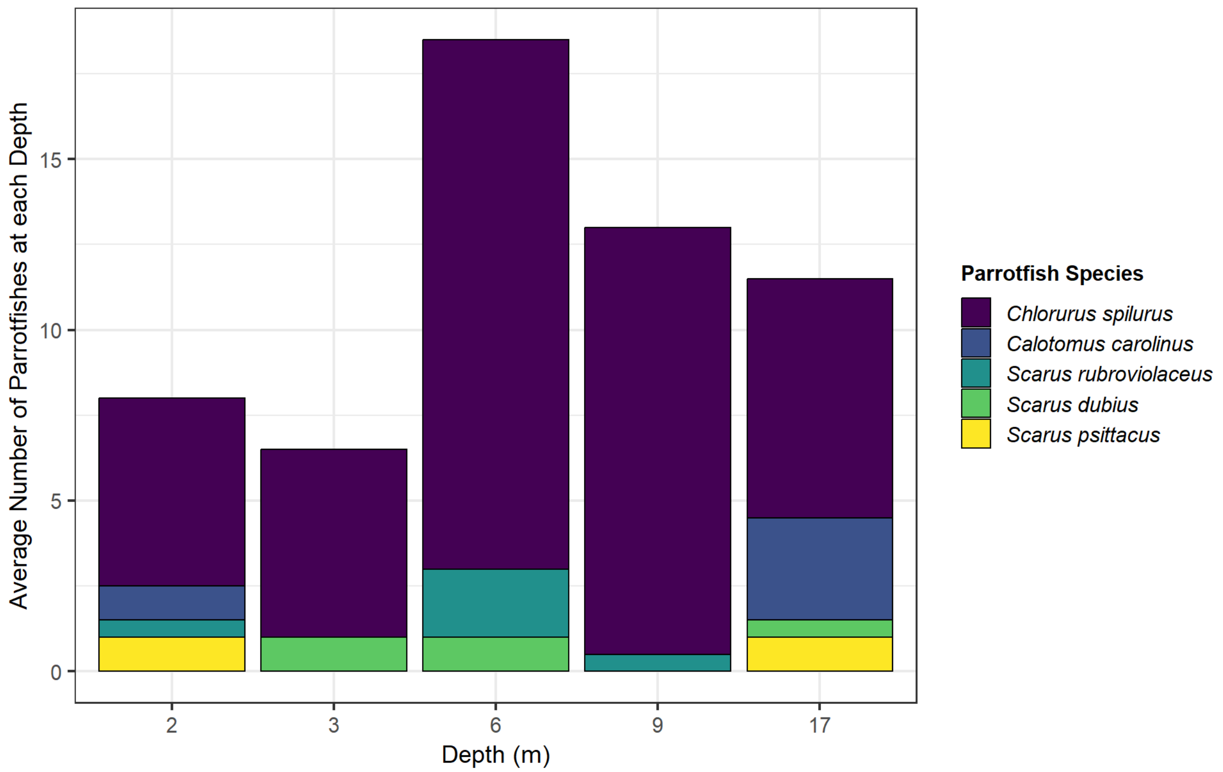

Fig M. Average number of parrotfishes by species surveyed in sixty 25 x 5 m belt transects at Sites 1 and 2 across depths (2–17 m) in Hōnaunau Bay, Hawai'i Island, 2023.

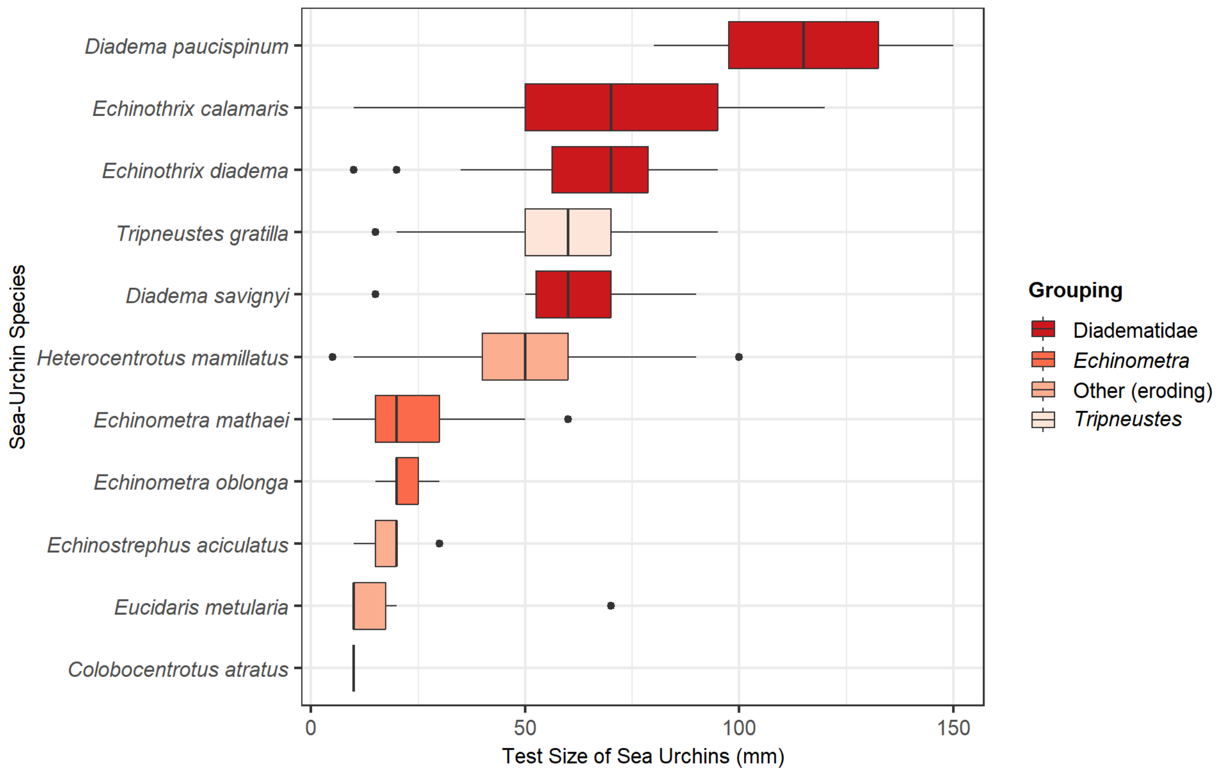

Fig N. Test (i.e., skeleton) size (mm) of sea urchins by species surveyed in 150 x 0.25 m² quadrats at Sites 1 and 2 across depths (2–17 m) in Hōnaunau Bay, Hawai‘i Island, 2023. The thick vertical lines indicate medians, the boxes indicate the first and third quartiles, the whiskers indicate the range of the data, and points indicate outliers. Diadematidae included species in the genera *Diadema* and *Echinothrix*, *Echinometra* included species only in the genus *Echinometra*, Other (eroding) included all other eroding sea-urchin species surveyed in this study, except for the non-eroding species *Tripneustes gratilla*.

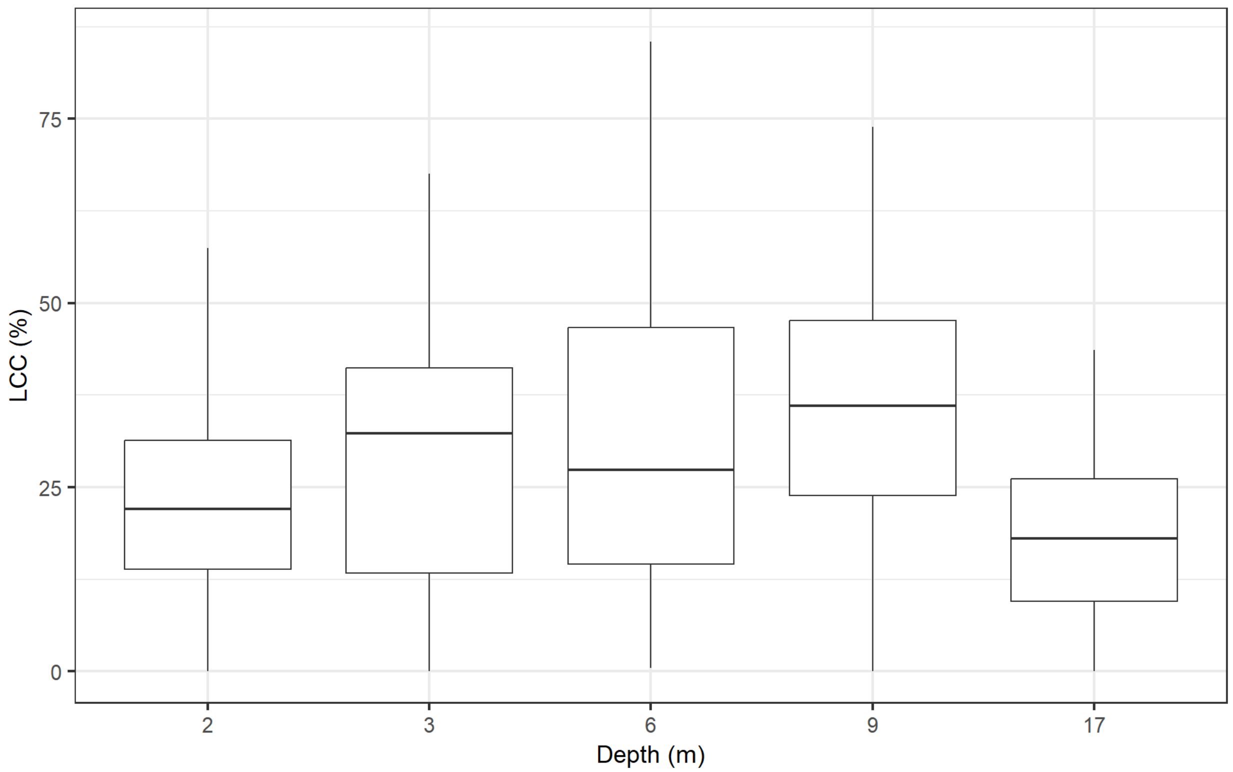

Fig O. Percent live coral cover (LCC) surveyed in 150 x 2 m transects at Sites 1 and 2 across depths (2–17 m) in Hōnaunau Bay, Hawai‘i Island, 2023. The thick horizontal lines indicate medians, the boxes indicate the first and third quartiles, and the whiskers indicate the range of the data. Note that outliers were removed from the display. A linear mixed-effects model and Tukey post-hoc test showed no significant differences in percent live coral cover among depths.

S1 File References

1. NOAA. NOAA Tide Predictions at 1617433 Kawaihae, HI. In: NOAA Tides & Currents [Internet]. 2024 [cited 19 May 2024]. Available: https://tidesandcurrents.noaa.gov/noaatidepredictions.html?id=1617433&legacy=1

2. Asner GP, Vaughn NR, Balzotti C, Brodrick PG, Heckler J. High-Resolution Reef Bathymetry and Coral Habitat Complexity from Airborne Imaging Spectroscopy. Remote Sens. 2020;12: 310. doi:10.3390/rs12020310

3. Asner GP, Vaughn NR, Foo SA, Shafron E, Heckler J, Martin RE. Abiotic and Human Drivers of Reef Habitat Complexity Throughout the Main Hawaiian Islands. Front Mar Sci. 2021;8: 631842. doi:10.3389/fmars.2021.631842

4. van Woesik R, Cacciapaglia CW. Keeping up with sea-level rise: Carbonate production rates in Palau and Yap, western Pacific Ocean. PLOS ONE. 2018;13: e0197077. doi:10.1371/journal.pone.0197077

5. Perry CT, Lange I, Januchowski-Hartley FA. ReefBudget Indo Pacific: online resource and methodology. 2018. Available: http://geography.exeter.ac.uk/reefbudget/

6. Grigg RW. Depth limit for reef building corals in the Au'au Channel, S.E. Hawaii. Coral Reefs. 2006;25: 77–84. doi:10.1007/s00338-005-0073-6

7. Brock RE. Species Profiles: Life Histories and Environmental Requirements of Coastal Vertebrates and Invertebrates Pacific Ocean Region; Report 5, The Parrotfishes, Family Scaridae. US Army Corps of Engineers; 1991.

8. NOAA. Relative Sea Level Trend 1617433 Kawaihae, Hawaii. In: NOAA Tides & Currents [Internet]. 2024 [cited 19 May 2024]. Available: https://tidesandcurrents.noaa.gov/sltrends/sltrends_station.shtml?id=1617433
